# Loss of pollinator specialization revealed by historical opportunistic data: Insights from network-based analysis

**DOI:** 10.1371/journal.pone.0235890

**Published:** 2020-07-13

**Authors:** Floriane Jacquemin, Cyrille Violle, François Munoz, Grégory Mahy, Pierre Rasmont, Stuart P. M. Roberts, Sarah Vray, Marc Dufrêne

**Affiliations:** 1 Biodiversity and Landscape, Gembloux Agro-Bio Tech, University of Liège, Gembloux, Belgium; 2 CEFE, Univ Montpellier, CNRS, EPHE, IRD, Univ Paul Valéry Montpellier 3, Montpellier, France; 3 Laboratoire d’Ecologie Alpine, Université Grenoble Alpes, Grenoble, France; 4 Laboratoire de Zoologie, Université de Mons, Mons, Belgium; 5 Centre for Agri-Environmental Research, School of Agriculture, Policy and Development, University of Reading, Reading, England, United Kingdom; 6 Département de Géographie, Université de Namur, Namur, Belgium; University of the Balearic Islands, SPAIN

## Abstract

We are currently facing a large decline in bee populations worldwide. Who are the winners and losers? Generalist bee species, notably those able to shift their diet to new or alternative floral resources, are expected to be among the least vulnerable to environmental change. However, studies of interactions between bees and plants over large temporal and geographical scales are limited by a lack of historical records. Here, we used a unique opportunistic century-old countrywide database of bee specimens collected on plants to track changes in the plant-bee interaction network over time. In each historical period considered, and using a network-based modularity analysis, we identified some major groups of species interacting more with each other than with other species (i.e. modules). These modules were related to coherent functional groups thanks to an *a posteriory* trait-based analysis. We then compared over time the ecological specialization of bees in the network by computing their degree of interaction within and between modules. “True” specialist species (or peripheral species) are involved in few interactions both inside and between modules. We found a global loss of specialist species and specialist strategies. This means that bee species observed in each period tended to use more diverse floral resources from different ecological groups over time, highly specialist species tending to enter/leave the network. Considering the role and functional traits of species in the network, combined with a long-term time series, provides a new perspective for the study of species specialization.

## Introduction

Insect pollinators have an essential ecological role and provide key ecosystem services [1,2]. The populations of many pollinators have been regressing worldwide for several decades [3]. In Western Europe, more intense agriculture and increasing urbanization resulted in drastic land cover changes and in a loss and fragmentation of natural habitat since the 1960s [4]. These habitat changes have had direct and indirect effects on bees, especially through a decrease in the availability of floral resources [5]. The consequences of these anthropogenic impacts have often been analysed from a taxonomic perspective [6,7], without taking into account the impacts on ecological interactions such as those that exist between plants and wild bees. However, the loss of interactions often occurs in parallel, or even precedes, the loss of species they involved [6,8].

Some bee species are essentially dependent (i.e. specialist) on a particular habitat, making them the first victims in the event of alteration or disappearance of this habitat. Resource specialization broadly varies among bees [9], so that forage specialists, which forage on one or a few plant taxa, appear to be more vulnerable than generalist species, which feed nonspecifically [10,11]. Generalist bee species especially seem more resilient in fluctuating environmental conditions when they are able to shift to exploit alternative resources (i.e. opportunistic behaviour) [8,12]. Floral specialization, classically estimated by the number of plant species visited, has been most often regarded as constant over time. However, some authors, such as Barnagaud et al. [13] in a study on birds, suggested that trophic specialization can in fact vary. Studies of these aspects in bee communities are still needed to improve understanding of their dynamics and their vulnerability over time [12].

The ecological interactions in which the species are involved condition their resilience, resistance and robustness to environmental changes (e.g. [14,15]). In the case of mutualistic interactions between plants and pollinators, it has been shown that some of them are more vulnerable than others and that non-random losses of ecological interactions can precede species disappearance [6,8]. In order to understand better the threats hanging over bee species and some response patterns (e.g. generalists *vs* specialists) to anthropogenic disturbances, it therefore seems pivotal to consider their interactions with plant resources. A simplified relevant approach to address these dynamics is the binary network analysis approach [16]. It represents interacting species as forming two types of nodes (i.e. plant and pollinator species), and the interactions between pollinator and plant species as links. The network is “binary” (*vs* “weighted”) when all interactions have the same weight (the value is 1 in the plant*pollinator contingency matrix when an interaction was observed, 0 otherwise). Classically, specialization is evaluated at species level through the species degree, i.e. the number of species with which the given species interacts, and at network level through the connectance, i.e. the proportion of observed interactions to all possible interactions [17–19]. However, these indices are not bounded and are known to be widely dependent on the size of the network and the sampling [17]. More innovatively, these aspects of species specialization can be studied through the degree of interactions within and between some functional groups identified within a network. Recent studies have demonstrated a non-random structure of pollinator-plant networks (e.g. [20,21]). Specifically, they identified the modularity property of networks made of subgroups of species (i.e. modules) interacting more between them than with others [22,23]. These preferential associations suggest re-evaluating species trophic specialization at the level of the modules representing functional groups with distinctive ecological properties [15]. The frequency with which a bee species interacts with different modules should reflect its ability to interact with species of diverse functional groups, which is not possible to identify with a classic approach of specialization analysis. This frequency can be broken down into two indices (“connectivity” and “participation” coefficients) representing different roles of species in the network. They correspond to the degree of interaction of a species within its module, or functional group, relatively to its interactions with species of other modules. They can then be used to assess the degree of species specialization toward the functional groups [24]. This way, a species that interacts with only few species from its own module is more specialist than a species that interacts with many species from several modules. Such an approach, based on network modularity analysis and species traits, is particularly relevant to test the hypothesis of different responses of functional groups to environmental change and to identify more precisely at which level specialization does change [15,25]. Depending on its role (or position) in the network structure, the disappearance of a species from the network can more or less affect the others. In particular, the loss of generalist species occupying a key role in their module and/or the network by connecting some modules is more likely to cause cascading extinctions [26] and threaten the survival of its partners [27].

Long-term studies are needed to address how the disruption of interactions can alter the persistence of partner species and the stability of plant-pollinator network [28,29]. However, few studies focused on the long-term lability of host-plant specialization in pollinator communities (i.e. interspecific variability) or populations (i.e. intraspecific variability) ([10]; but see [29,30]). Due to a lack of historical data on interactions observed in the field (but see [29]), most surveys of plant-pollinator networks have been limited in temporal extent [28], or resorted to simulation experiments to extrapolate long-term trends [31]. Some studies examined parallel changes in pollinator communities and historic dynamics of vegetation or land cover/use (e.g. [10,32]). Other studies retrospectively analysed pollen collected on museum specimens, identifying effective interactions between plants and their pollinators, but they focused on a limited number of bee species (e.g. from the genus *Bombus* [12,33] or *Andrena* [34]) and/or did not consider a network approach [35]. In this context, opportunistic data, i.e. data collected by volunteers without following a precise protocol can be useful. Despite their limitations in sampling accuracy, these often-massive datasets have the advantage of covering large areas and/or time periods [36], which can help identify hidden patterns of interaction network dynamics.

In this study, we investigated a century of changes in bee-plant interactions in the southern part of Belgium, where bees are drastically declining (e.g. [37,38]). Landscapes in this country are representative of massive land use dynamics that has happened in western Europe during the last century and should have particularly affected plant-pollinator networks. We analysed a unique historical database of opportunistic geo-referenced floral visit records at the Belgian continental bioclimatic region scale on which no network analysis has yet been performed. We compared networks built on observations made during two large time periods in order to reduce the noise from the short-term natural turnover in community composition [30,39], to overcome subsampling, and thus to highlight large shifts that may have appeared over time. Scaling-up local interactions to a large-scale pollination network can help identify consistent patterns of plant-pollinator interactions across a biogeographical area [40,41], revealing species specialization over distinct ecological contexts. More specifically, we explored changes in network structure over time through the specialization of species within and between modules. We analysed the composition of the different modules using species’ functional traits to investigate their ecological nature [42]. We expected that (i) generalist species have been less impacted by anthropogenic disturbances and that their frequency increased over time in the interaction network and that (ii) species observed in both ancient and recent periods diversified their diet. To go deeper into the underlying causes, we tested how candidate ecological traits of bee species are associated to specialist *vs* generalist strategies (e.g. phenological traits like flight period duration or morphological traits like body size), which represents a long research avenue for pollination ecology [43–45,22].

## Material and methods

### Study site and interaction dataset

Belgium is representative of the lowland agricultural landscapes of western Europe, deeply modified by increasing agricultural intensification and urbanization during the last century [46,47]. The targeted area is the continental bioclimatic region of Belgium [48], where we had homogeneous distribution of bee occurrence data (S1 Fig in S1 Appendix). Although the plant-bee interaction data were less well dispersed, the main modules identified (calculation: see below) were distributed over the whole area and did not correspond to territorial boundaries depending on the sampling (S1 Fig in S1 Appendix).

We obtained interaction data from a national database called “*Banque de Données Fauniques de Gembloux & Mons*” (“BDFGM”, established under the EU FP7 STEP project, see [49] for more details), which includes more than 200,000 bee specimen occurrence data, including 14,000 bee specimens (203 species) recorded on plant species by naturalists in the study area between 1930 and 2010 (S1 Fig in S1 Appendix). All these opportunistic data were validated by experts.

We compared two historical periods, 1930–1969 and 1990–2009 (see S2 Fig in S1 Appendix for detailed information about their selection), which represent contrasted contexts related to major economic and land-use changes, i.e. during and after the main intensification of agriculture, respectively [50]. For each period, we built a bipartite network of observed bee-plant interactions. We included only the interactions observed twice during the period considered between taxa identified at the species level, to avoid possibly spurious singletons. We then built the binary adjacency matrices, i.e. the matrices of interaction occurrences between plants and bees (Table 1; S1 Table). We did not weight the matrices by the number of bee specimens observed per plant species per period, as it could have been over- or underestimated according to the sampling method used.

**Table 1:** Network dimensions according to historical periods (1930–1969 and 1990–2009).

Dimensions	1930–1969	1990–2009	1930–2009	Rich. tot
Number of bee species	132	126	68	190
*Families*	6	6	6	6
*Genera*	23	24	21	26
Number of plant species	202	206	92	316
*Families*	42	42	34	50
*Genera*	144	137	84	197
Number of interactions	541	795	66	1270

The column “1930–2009” considers the number of species and interactions observed in both periods. The “total richness” shows the number of unique species and interactions observed over time.

The honeybee (*Apis mellifera*) was not included because its presence is largely related to human activities [51]. Conversely, cleptoparasites were taken into account since we looked at broad scale visitation rather than either pollen or nectar foraging *per se* [52].

### Network analysis

#### Calculation of classical specialization indices

Specialization at species level in binary bipartite networks is classically quantified as the degree of each species, i.e. the number of species from the other group with which they interacted. In case of trophic interactions, this specialization can be considered as a proxy for trophic niche breath [17], so that the higher this index, the more generalist a species [53]. To calculate the degree of bee species, we used the “*specieslevel”* function from the R package “*bipartite*” [54] and tested the significance of difference of species degree between periods using a Wilcoxon test. At the network level, we calculated the connectance index defined as:

$$C=\frac{N}{p*b}$$ (1)

with p = number of plant species, b = number of bee species and N = the total number of observed interactions [55]. This index was computed using the “*networklevel”* function from the R package “*bipartite*” [54]). To test the significance of difference in connectance between periods, we compared the distributions of connectance of matrices rarefied 1000 times, with a fixed total richness (i.e. number of plant plus pollinator species) [56]. The dimensions of rarefied matrices corresponded thus to the sum of the minimal dimensions of observed matrices (i.e. *min*(observed number of bee species) + *min*(observed number of plant species) = 126+202 = 328; Table 1). We compared the distributions of the connectance values for the rarefied matrices between periods using a Wilcoxon test.

#### Analysis of modularity

The other approach we used was to characterize species specialization in the network based on how their interactions were distributed within and across modules of the network at each period. We delineated the modules with the method proposed by Guimerà and Amaral [24] for bipartite networks, using simulated annealing (R package “*rnetcarto*”; [57]). The analysis yielded groups of bees (i.e. modules) based on the floral resources they share [22] and such as the interaction density within modules is higher than between modules [20,58]. It implied that bees had a majority of their links inside their own module with an accuracy of 90% [59]. The modularity statistic is equal to 0 for randomly configured networks, and peaks to 1 for networks composed of completely separated modules. We assessed the significance of modular structure by period by comparing observed modularities to null values in networks where the links were shuffled, while the number of links of each node was kept constant as in the observed network ("*independentswap*" algorithm, R package *picante*; [60]). We calculated the Standard Effect Size in order to standardize the measure of the deviation of the observed modularity from those calculated for null models such that:

$$SES=\frac{M_{obs}-{\bar{M}}_{null}}{{SD}_{null}}$$ (2)

with *M_obs_* = observed modularity, ${\bar{M}}_{null}$ = mean modularity of null models and *SD_null_* = standard deviation of null models’ modularity. A *SES* above 1.96 indicates significantly modular network structure with a risk of error of 5% [61]. In addition, we evaluated the significance of the difference of modularity between the two periods using Wilcoxon test comparing the distributions of differences between observed and null modularities (package *stats*; [62]). To test the consistency of the results, we resampled 1000 times the second matrix (1990–2009) with the R function “*sample*”, to fix the number of interactions to the one of the first period (1930–1969), and compared the observed modularity of the first period to the distribution of modularities calculated for resampled networks of the second period.

#### Evaluation of species specialization through the calculation of connectivity and participation coefficients

With regard to the characterization of the role of species and their specialization in the network, we relied on Olesen et al. [20] who proposed two descriptors to characterize inter- and intra-module connectivity (*c-* and *z*-coefficients, respectively), based on [24,59]. The *c*-coefficient (“participation” coefficient) describes the level to which the species is linked to species from other modules. The *z*-coefficient (“connectivity” coefficient) describes the standardized number of links to other species in the same module. Following Olesen et al. [20], “peripheral” species, i.e. species displaying low *c-* and *z-*values, could be considered as specialists. These species are involved in only a few links and almost always only with species within their module. “Hub” species, i.e. species with high *c-* and *z*-values, could be considered as generalists. They include “module hubs”, i.e. highly connected species linked to many species within their own module (low *c-* and high *z-*coefficients), and “connectors”, i.e. species that link several modules (high *c-* and low *z-*coefficients). Species with both a high *c-* and *z-*coefficients are “network hubs” or “super generalists”. We considered the key species role (i.e. module hub, connector and network hub) as significant when *cz*-coefficients were higher than thresholds corresponding to the 90% quantiles of *cz*-coefficients of species from null models. We also tested the 95% threshold to be more conservative, following Dormann & Strauss [63]. These *cz*-indices were calculated for 124 bee species before 1970, and 121 species after 1990 (when the denominator of *z*-coefficient was not null, i.e. when the variance of the interaction number in the module of species belonging to this module was not null).

To test the hypothesis (i) that generalist species have been less impacted and therefore their relative frequency increased over time, we evaluated the significance of the difference of *cz*-values between the two periods using Wilcoxon tests. To test the hypothesis (ii) that species observed in both ancient and recent periods diversified their diet, suggesting an opportunistic behaviour, we focused on the 64 bee species observed in both time periods. We performed a one-tailed paired Wilcoxon test to assess whether the *cz*-coefficients of bees tended to become higher after 1970. In the case of species that appeared (disappeared) in the network after 1970, we tested if their *cz*-coefficients were higher (lesser) than that of other species in the same period with one-tailed Wilcoxon tests.

Finally, we compared these *cz-*values to species degrees to test the difference between this approach and the classical one. Although one can expect a link between the *cz*-values and these features (e.g. Biella et al. [64] found that the higher the degree of a species, the more likely it is to be a hub in the network), the classical approach ignores some aspects of species specialization related to the role of species in the network structure. We calculated the correlation between degrees and *cz-*values using Pearson coefficients.

#### Trait-based characterization of modules

We identified modules for which the composition remained similar from one period to the other by performing a single hierarchical clustering analysis of bee species*module matrices. We used Jaccard distance (R function “*vegdist*” from the package “*vegan*”) and Ward’s clustering method (R function “*hclust*” from the package “*stats*”). We checked the taxonomic homogeneity within the main modules (i.e. containing ≥ 10 bee species at each period) in terms of plant species composition by calculating the plant species-by-genus ratio of each module over the two periods (following Elton [65]), i.e. number of species/number of genus per module. We compared the observed values to those calculated for the null models built above. The higher the ratio, the more taxonomically homogeneous the module.

To explore the ecological coherence and identify the main characteristic traits of the modules identified in the networks, we described the functional module composition by integrating trait data of the interacting species that are known to intervene (among others) in their interactions [66].

To compare bee species’ foraging behaviour according to modules, we extracted trait data linked to foraging strategies from a database built as part of the EU FP6 ALARM and EU FP7 STEP projects and completed it with data based on a broad sweep of European bee literature and on researcher expertise (e.g. [49,67,68]). We selected six species traits: mean intertegular distance (ITD = distance between the wing bases in mm; available for 184 species), used as a proxy for body size [69]; tongue length (two categories: short or long; for 188 species) [70]; sociality (six categories from most to least social traits: primitively eusocial, i.e. live in small colonies in which the females go through a solitary phase or may switch between roles as workers or queens; communal, i.e. females share a common nest entrance but supply their own cells, solitary+primitively eusocial, i.e. have a polymorphic sociality; solitary, i.e. all females are fertile and individually build their nest; social parasite, i.e. mated females or workers are adopted by host species colonies; and cleptoparasites, i.e. lay their eggs in nests built and supplied with pollen by host species; for 187 species) [71]; duration of the flight period (four categories: spring, summer, spring-summer, and the entirety of the favourable season; for 188 species) [44,64]; nesting behaviour (five categories: carder, i.e. surface nesters using shredded plant material; excavator, i.e. digging holes in the ground; renter, i.e. using existing cavities; parasites, i.e. species using nests of other bee species; mason, i.e. using mud to fashion entire cell; for 188 species) [52] and lecty trait (two categories: oligolectic, i.e. visiting a small number of floral genera from a single plant family; and polylectic, i.e. visiting several floral genera from more than one plant family; for 188 species) [72]. We used these traits representing bee species’ foraging behaviour to characterize the functional trophic nature of modules and the relationship between trophic strategies and specialization. In the later case, we performed Wilcoxon test and quantile 30% regression to compare *cz-*values (see below for their calculation) and ITD trait. We also used Kruskal-Wallis tests to compare *cz-*values between categories of sociality, lecity, nesting behaviour and duration of the flight period. We calculated the proportions of traits presented by bee species included in the main modules.

We also compiled trait data of plants included in the networks to identify the characteristics of plant resources in main modules. We extracted traits from [73], [74] and the BiolFlor database [75]. We focused on flower morphology and plant phenology, as they are expected to mediate interactions with pollinators [66], and because they are available for a maximum number of species. The traits retained were: flowering duration (four categories: 1–2 months, 3–4, 5–6, >6; for 307 species), a longer flowering period potentially allowing the visit by more bee species [76], and flower type (eight categories: disk, heads (Asteraceae and non-Asteraceae), lip, stalk disk, bell, flag, funnel, brush; for 269 species) based on Kugler classification [77], the depth of the corolla having a link with the type of visiting bee (tongue length) [78]. We calculated the proportions of traits presented by the plant species interacting with bees included in the main modules.

All data analyses were performed with R 3.3.1.

## Results

We found changes in network structure over time. Although the number of interacting species was quite similar, the number of interactions was higher between 1990–2009 than between 1930–1969 (Table 1). Almost 30% of the plant species were observed in both periods, representing ca. 45% of plant species observed per period. More than 35% of the bee species were identified in both periods. Less than 10% of unique interactions between a plant and a bee species were maintained across both periods.

As part of the species specialization analysis based on classical indices for binary networks, we found that the mean value of bee species degree increased from 4.24 before 1970 to 6.51 after 1990 (but not significantly: p = 0.214). At the network level, the connectance increased from 0.020 before 1970 to 0.031 after 1990. The difference between periods of the distributions of the connectance values calculated for the rarefied matrices was significant (p < 0.001). Network modularity decreased significantly over time (M = 0.475, SES = 10.48 before 1970; M = 0.279, SES = 9.27 after 1990, p < 0.001). We resampled the matrices to control for the number of interactions, and we still found a decrease in modularity over time (S3 Fig in S1 Appendix). The number of modules was similar between periods (11 and 10 modules with less than two percent of species distributed in very small modules and/or unclassifiable). Based on the clustering analysis, we identified that three main modules (≥ 10 bee species) were consistent at both time periods (S4 Fig in S1 Appendix; Table 2). Two of them were mainly composed of short-tongued bees: one mainly composed of Halictidae species (61% on average of bee species in the module), interacting mainly with plant species with head flowers such as Asteraceae (30% on average of interactions), and the second one mainly composed of Andrenidae species (60% on average of bee species) mainly interacting with plant species with disk flowers such as Rosaceae (22% on average of interactions). The flowering period of plants in the later module was significantly shorter than in the two other modules. The last module contained mainly long-tongued bees: Apidae species (64% on average of bee species) interacting mainly with plant species with lip and flag flowers such as Lamiaceae and Fabaceae (25 and 15% on average of interactions, respectively). This within-module homogeneity for plant composition was confirmed by a higher species-by-genus ratio than expected by chance (S5 Fig in S1 Appendix).

**Table 2:** Characteristics of the three main modules (containing ≥ 10 bee species) in terms of the traits of bees and plants with which they interact.

		Module N°		
		1	2	3
**Bee family**		Halictidae (61%)	Andrenidae (60%)	Apidae (64%)
Bee traits	**Mean ITD (mm)**	1.92	2.32	3.74
	**Tongue length**	Short (79%)	Short (79%)	Long (90%)
	**Flying period duration**	Summer (45%)	Spring (60%)	Year (44%)
	**Lecty**	Polylectic (62%)	Polylectic (88%)	Polylectic(83%)
	**Nesting behaviour**	Excavator (68%)	Excavator (84%)	Renter (50%)
	**Sociality**	Solitary (42%)	Solitary (74%)	Solitary (47%) & Eusocial (39%)
**Flower family**		Asteraceae (30%)	Rosaceae (22%)	Lamiaceae (24%)
Plant traits	**Flower type**	Heads (43%)	Disk flowers (57%)	Lip flowers (25%)
	**Flowering period duration**	3–4 months (58%)	1–4 months (85%)	3–4 months (60%)

The maximum average proportions of qualitative traits over the two periods are indicated between brackets.

**Fig 1:**
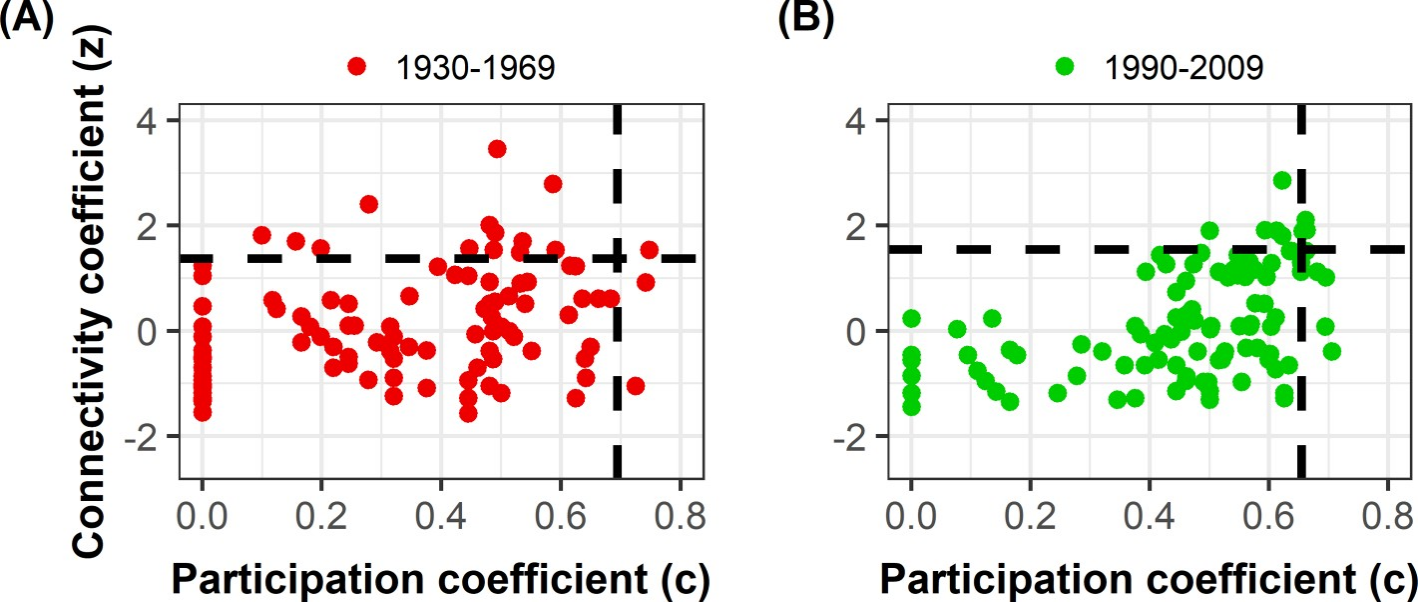
Biplot of connectivity coefficient of bee species (*z*, ordinates) and their participation coefficient (*c*, abscissa). (A) *cz*-values of bee species before 1970 and (B) after 1990. Vertical and horizontal dashed lines represent 90% quantiles of null model coefficients and delimit groups of species with different topological roles in networks [24,59,79].

**Fig 2:**
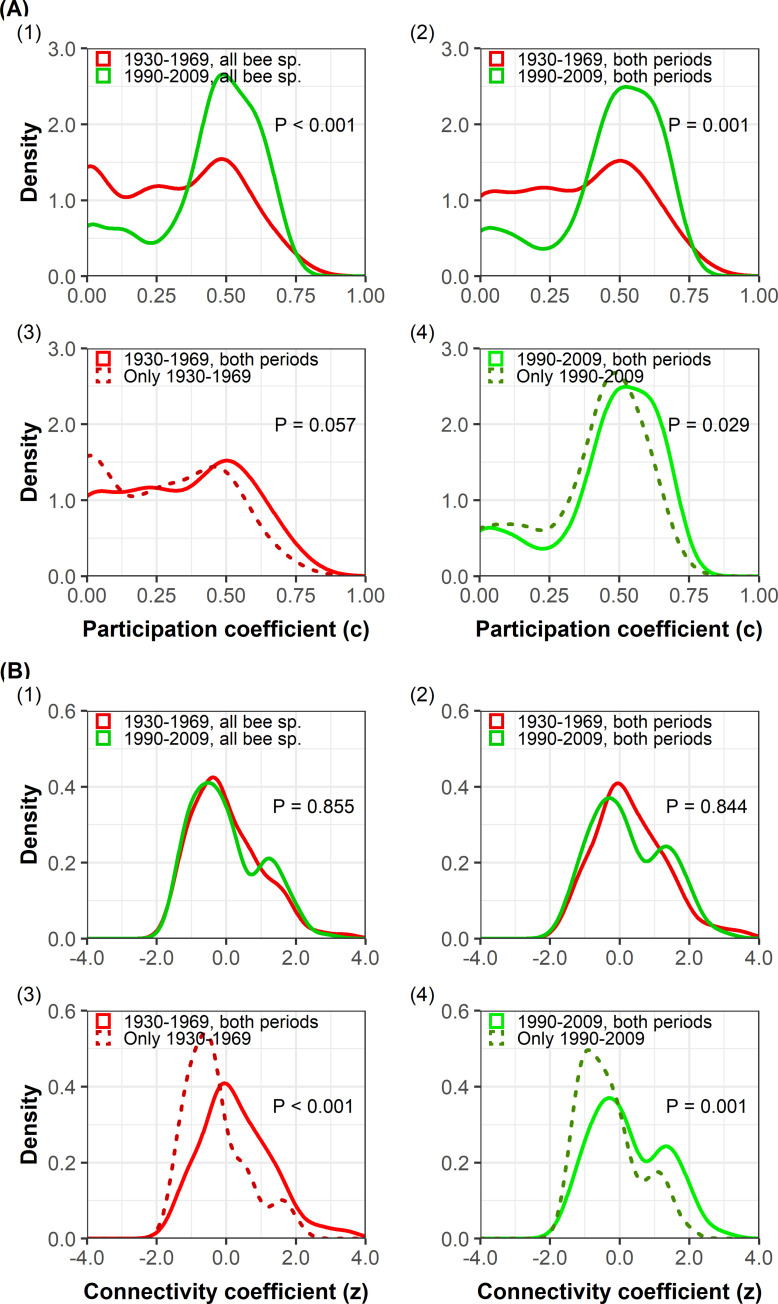
Distribution of participation coefficient (*c*) and connectivity coefficient (*z*) of bee species. Distribution of *c*-coefficient (panel A) and *z-*coefficient (panel B) of (1) all bee species per period (red: 1930–1969; green: 1990–2009); (2) bee species persisting in the network (i.e. observed during the two periods) per period (red: 1930–1969; green: 1990–2009), (3) bee species that disappeared from the network (dashed line) and persisting species (solid line) between 1930 and 1969, (4) bee species that appeared in the network (dashed line) and persisting species (solid line) between 1990 and 2009 [24,59,79].

The *c*-coefficient of bee species increased over time (mean *c =* 0.30 before 1970 and 0.43 after 1990, p < 0.001), while their *z*-coefficient did not vary (p = 0.855) (Figs 1 and 2). We observed in particular that the *c-*coefficient of the 64 bee species present in both periods increased significantly over time (mean *c* = 0.33 before 1970 and 0.45 after 1990, p = 0.001; Fig 2A; S6 and S7 Figs in S1 Appendix) but not the *z-*coefficient (mean *z* = 0.30 before 1970 and 0.26 after 1990, p = 0.844; Fig 2B). Almost 60% of these species had a higher *c-*coefficient after 1990 than before 1970, and thus tended to become more generalist over time. On the other hand, the *c*z-coefficients of the 60 species that were no longer observed in the network after 1990 were lower than those of other species (mean *c-*coefficient of species disappearing from the network = 0.26 *vs* 0.33 for other species, p = 0.041 and mean *z* = -0.32 *vs* 0.30, p < 0.001; Fig 2), indicating a loss of more specialist species. Likewise, the *cz-*coefficients of the 57 species that were observed only in the second period were significantly lower than the *cz-*coefficients of other species (mean *c* of species appearing in the network *=* 0.41 *vs* 0.45 for other species, p = 0.029 and means *z* = -0.29 *vs* 0.26, p = 0.001; Fig 2).

Using 90% quantiles as thresholds to define significant roles, we identified 17 key species before 1970 (i.e. 14 module hubs and three connector species) and 13 key species after 1990 (i.e. five module hubs, three network hubs and five connectors) (S2 Table). Some species having a key role before 1970 were not observed in the network or became peripherals after 1990 (e.g. *Andrena bicolor*). Other species that were peripherals or not recorded before 1970 became module hubs or connectors after 1990 (e.g. *Chelostoma rapunculi*). Five species kept a key role in the networks in both periods (e.g. *Bombus pascuorum*). The 17 key bee species identified before 1970 and the 13 key species identified after 1990 interacted with 113 and 172 plant species, representing 56% and 83% of observed plant diversity, respectively. The plant species with which they have the highest degree of interaction are listed in S2 Table.

Using the 95% quantiles of *cz-*coefficients, we identified six key species before 1970 (four module hubs, one network hub and one connector); and four after 1990 (one module hub, one network hub and two connectors) (see S2 Table for more details).

We calculated the correlation between the *cz-*coefficients and the classically calculated index at the species level, i.e. the species degree. This index was significantly correlated with *cz-*coefficients (p < 0.001, Pearson correlation index = 0.45 between *c* and degree and 0.61 between *z* and degree; S8 Fig in S1 Appendix).

**Fig 3:**
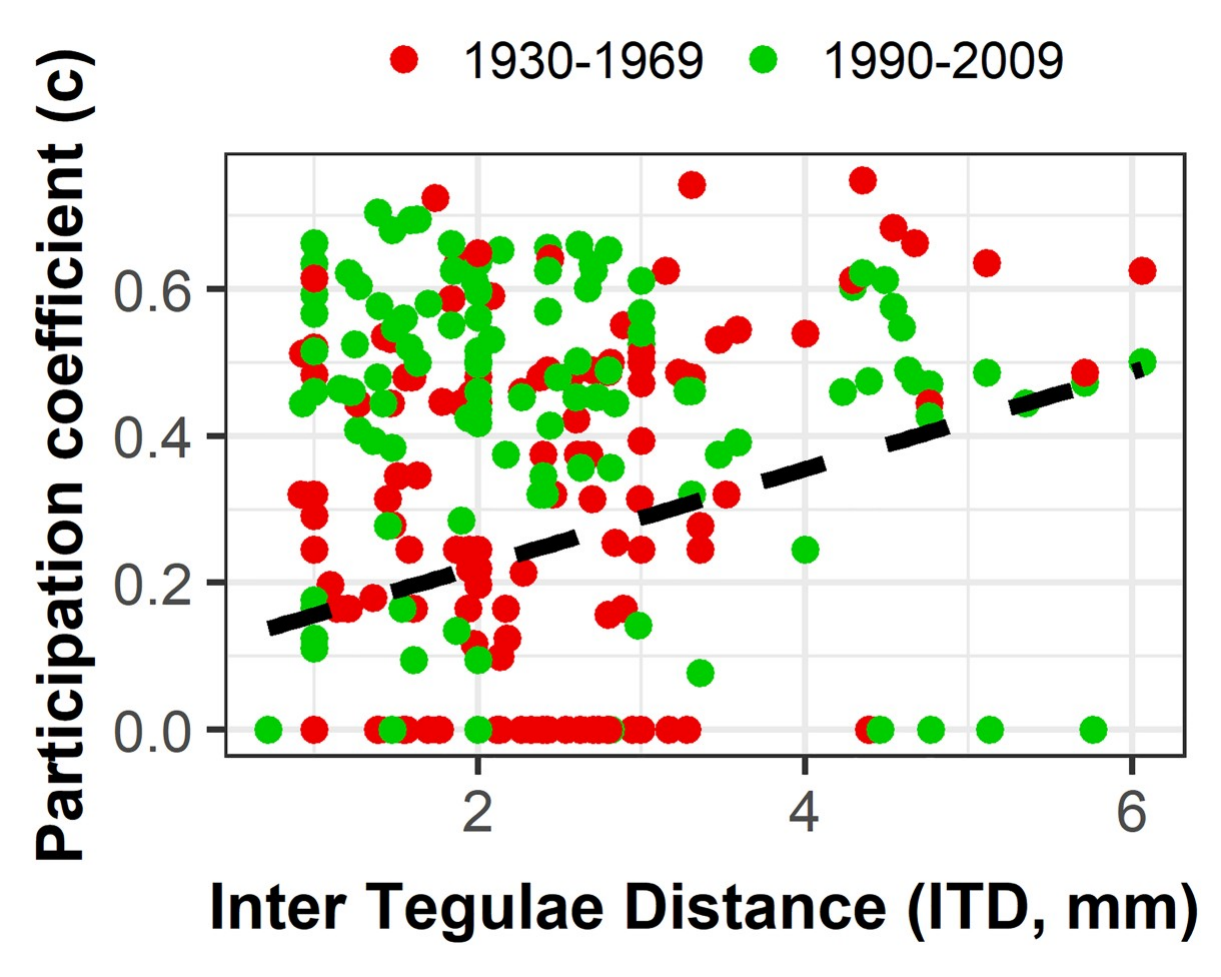
Distribution of intertegular distance according to participation coefficient of bee species. Intertegular distance (ITD, mm) of bee species according to their *c*-coefficient by period (red: 1930–1969; green: 1990–2009). The dashed line is a quantile regression line (30%).

Species with a higher *c*-coefficient tend to be polylectic species that fly throughout the favourable season (p < 0.05) and/or have a larger body size (Fig 3). We did not find any significant relationship between the *c*-coefficient and other bee traits. Almost 90% of key species (i.e. hubs and/or connectors) are polylectic.

## Discussion

By analysing the long-term dynamics of a plant-bee interaction network at a large geographical scale, we found a high turnover of bee species and of their interactions with plants over time. Only 35% of species, and less than 10% of unique interactions, were observed before 1970 and after 1990. Such a temporal variability was also observed in other studies (e.g. [28,34]). In Belgium, it can be related to the land use changes induced by agricultural intensification and urbanization that have also deeply impacted biodiversity in Europe [35].

The analysis of the structure of the plant-bee network modularity highlighted three main modules whose composition was globally homogeneous at a higher taxonomic level (families of bees visiting more or less the same families of plants) and in terms of bee and plant species traits (long tongued bees visiting plants presenting flowers with long corolla and *vice versa*). Apart from compositional changes and plasticity of species interactions, these fundamental functional groups of bees and plants stayed robust to environmental changes. This trait-based approach further demonstrates the relevance of the modules, especially from a functional point of view. Modules conserved their homogeneity and ecological relevance over time. When they are available, some other traits should be included in the analysis to refine the definition of the functional groups identified, e.g. the colour of flowers is known to have an important effect on the foraging behaviour of bees [80].

We quantified the specialization of individual species firstly through the calculation of classical indices. The analysis of the connectance revealed a tendency towards a decrease in specialization at the network scale over time while the analysis of species degree did not highlight any significant change of specialization at the species level. Secondly, we considered their degree of interaction inside (*via* the *z-*coefficient) and between (*via* the *c-*coefficient) modules, to compute their *cz*-coefficients as surrogates for their functional specialization. Although these coefficients are partly correlated to species degree, we highlighted that they provide complementary insights on the nature of specialization at the level of consistent ecological groups [53,64]. Indeed, in addition to the number of interactions in which a species is involved, this method distinguishes species interactions within and between modules and considers that a species interacting with several modules, representing functional groups, is less specialist than a species interacting with only one group [24].

Based on the modularity analysis, we first found that bee species were globally subservient to their module with the same frequency over time since their *z-*coefficient did not change significantly between periods. It confirms the relevance of these ecological groups and their robustness from one period to another, despite the variations in individual species roles. Secondly, we found an overall increase in *c*-coefficient of bee species, associated to a decrease in network modularity. Therefore, although the intensity of their interaction within modules did not vary, there were increasing links across modules in the recent period, suggesting a decrease in specialization over time in the network. This was mainly related to the significant increase of the *c*-coefficient of persisting bee species (i.e. observed in the network during both periods), illustrating the increase in their generalism. Some of them were able to shift their diet to alternative resources and diversified their visits [12,34]. They interacted with more ecological groups in the recent period, potentially because their preferred functional group did no longer provide enough resources. Such an opportunistic behaviour, suggesting some plasticity in trophic strategy, may have contributed to their maintenance in the network despite profound habitat changes. This behaviour is however limited by the need to display the appropriate traits and learning abilities to balance costs and benefits of foraging on new plant resources [81,82]. Further analyses are necessary to identify the mechanisms inducing the visit of plants from new modules (e.g. insufficient abundance of some plant species or changes in other characteristics of the functional group).

Besides the group of bee species persisting in the network over time, some others were not observed during both periods compared. Based on the analysis of their *cz*-coefficient, significantly lower than for persisting species, these species that disappeared/appeared over time in the network were more likely to be specialists [33]. Specialist species are known to be more sensitive at exit and entry into the network [39]. The rarity and their lower detectability of some of them [39] may partly explain their turnover across periods and potentially reflect some sampling issues. Some of these specialist species could persist or appear in the network by interacting with generalist plants [83] or because their host plants were not negatively affected by anthropogenic disturbances [35]. Finally, some others disappeared plausibly because they were less able to switch their diet and were therefore more sensitive to habitat fragmentation [29,84] and changes in plant community composition [12,85], partly explaining the decrease in specialization in the network over time.

By comparing the *cz*-values of species to thresholds calculated using null models, we detected some generalist species occupying the key roles of hubs and/or connectors in the modular structure of the network. These thresholds are arbitrary but allow identifying the species with more extreme roles. All the other species have a secondary role in the structure and are considered to be peripheral [64]. The key roles in the network structure were not provided by all the same species between periods. The high key species turnover and the relative loss of hubs and connectors in the network over time could point to increasing network vulnerability. Some modules could be more prone to collapse than others by losing module hubs (due to species disappearance or changing role) and could contribute to decreasing network modularity. Further studies will be needed to determine the implications in terms of ecosystem functioning (i.e. are the new key species in the network functionally equivalent or are we facing a " functioning debt"?). Indeed, plant-bee interaction networks should greatly impact ecosystem functioning and services through their role in plant reproduction [86].

Some candidate traits appeared to be related to bee species specialization. We showed that the most generalist species had a larger body size, which can be associated with longer foraging distance [69], and a longer flight period than species limited to their module (i.e. with low *c*-coefficient). Thanks to these characteristics, these species can have access to higher resource diversity/quantity [43,87], even in a context of habitat fragmentation and changes in plant communities. These species are also known to be polylectic, which confirms the partial correlation between *cz*-coefficients and degree of species [64]. Note that the integration of other traits should allow to go further in understanding the species specialization-trait relationships and identify those favourable to an effective foraging in a disturbed landscape.

To ensure the viability and the functioning of plant-pollinator networks, mitigation strategies should focus on key species [64]. In our study, as we noticed that key species are actually mostly widespread species in Belgium (e.g. *Bombus lapidarius*, *B*. *pascuorum*, *Halictus tumulorum*) [12], we should at least ensure their long-term monitoring. Declining, rare and specialist species represent an undeniable heritage interest, have a high conservation value [88,64] and participate in plant pollination (although to a lesser extent than generalist species). However, if the objective of the conservation measures is to maximize this service and/or to maintain the functionality of the interaction networks, they should not be the priority targets [20].

Due to a lack of standardized historical and large-scale data, the interest of opportunistic data has been widely demonstrated and discussed in the literature [36,89,90]. They are increasingly used for estimating trends and geographic range sizes [36]. However, these data, collected in a non-structured way, are often biased in time (variation of sampling methods over time), space and according to collector preferences. In our case, we are aware that it could influence the structure of plant-pollinator networks [91] and especially have had an impact on the observed turnover of species and interactions. Depending on the data available and the research question, it may be interesting to consider shorter periods to better understand this turnover. Moreover, specialization tends to be underestimated when based on the analysis of binary interaction information [92] and should take into account interaction frequencies between plants and pollinators (i.e. weighted network analysis) [17,27,93]. For instance, a species involved in several interactions can be considered as “generalist” through a binary approach even if 90% of its interactions concern a unique partner species, the others being occasional. Apart from such limitation, our dataset mainly included data collected (or at least identified) by professionals, and their analysis at such a large time scale allowed to reveal large patterns of interaction dynamics, and particularly a loss of specialization previously highlighted in more local contexts. In addition, we limited both the influence of rare interactions in our analyses by applying a minimum threshold for the number of interactions, and the sampling bias by comparing the structure of the observed networks to that of null models [55].

In conclusion, our network- and trait-based approach demonstrated the interest of module analysis to uncover the inherent functional architecture of plant-bee interaction networks, and offers new perspectives for the assessment of species specialization [15]. In our case, the comparison of large temporal and geographical networks in a regional context, built on an opportunistic database, highlighted a global decrease in bee species specialization over the last century. Analysing the dynamics of interactions and species roles within and between functional groups in response to environmental disturbances is essential to better understand the threats to species [8] and ecosystem functioning [29]. Such considerations should come into play when evaluating the conservation status of species.

## Supporting information

S1 Appendix (PDF)

S1 Table Code, full name, family and degree of interacting plant and bee species per period (1930–1969 and 1990–2009). (PDF)

S2 Table Key bee species identified by comparing their *cz*-coefficients with thresholds corresponding to the 90% quantiles of *cz-*coefficients of null models. Module hubs has higher *z*-values and lower *c*, connectors has higher *c-*values but lower *z* and network hubs has both higher *c* and *z*. ^α^ = species that disappeared from the network after 1990; ^β^ = species that became peripheral after 1990; ^γ^ = peripheral species that became key species after 1990; ^δ^ = species that appeared in the network after 1990 as a key species; underlined = species that had a key role during both periods; in bold = species that kept their key role when we used the 95% quantiles of *cz-*coefficients of null models). Plant species with which they interacted the most before 1970 and after 1990 were the 10 species that have the maximum degree with key species.
(PDF)

S1 Data (ZIP)
