# Supplementary material for: Loss of pollinator specialization revealed by historical opportunistic data: Insights from network-based analysis
Source: PLoS One. 2020 Jul 13;15(7):e0235890. doi: 10.1371/journal.pone.0235890 (PMC7357768; doi:10.1371/journal.pone.0235890)
Supplement: S1 Appendix — (PDF) [file pone.0235890.s001.pdf]

SUPPORTING INFORMATION

Loss of pollinator specialization revealed by historical opportunistic data: insights from network-based analysis

Floriane JACQUEMIN, Cyrille VIOLLE, François MUNOZ, Grégory MAHY,
Pierre RASMONT, Stuart P.M. Roberts, Sarah Vray, Marc Dufrêne

S1 Fig. Distribution of data collected between 1930-1969 and between 1990-2009 in the continental biogeographical region in Belgium. Left: data of bee specimens identified at the species level in the continental biogeographical region (dark grey; extreme coordinates are 50°49'38,97" ; 5°37'0,36" North, 50°20'5,21"; 6°25'6,54" East, 50°08'8,25"; 4°08'16,22" West and 49°29'49,05"; 5°28'56,26" South), used to define periods (1930-1969 in red, 1990-2009 in green). Right: data used for the network analysis (interactions between bee and plant species; minimum two bee specimens recorded per interaction per period). Although these data are relatively clustered, the three main modules identified did not correspond to geographical regions, allowing us to consider that this clustering of data has a limited effect on the results.

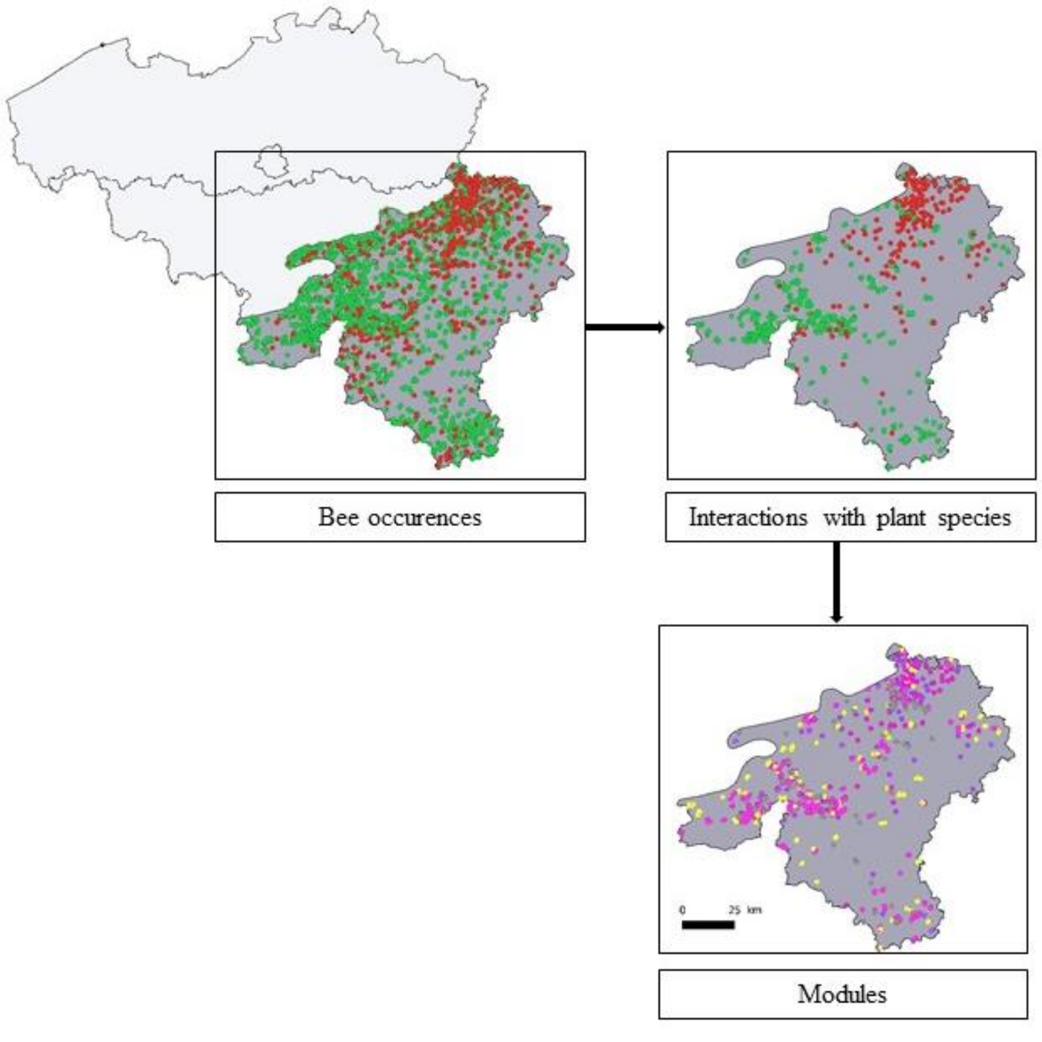

Selection of periods (1930-1969 & 1990-2009)

To define periods to compare, we split the bee occurrence data (S1 Fig) into 20-years periods to identify potential differences in terms of bee composition (and to check if these differences could be also found at the level of their interaction network with plant) and then define a pivot date. Since the period 1930-1949 and 1950-1969 has similar composition, we considered them together.

Then we compared the dimensions of the interaction networks with plants of each of these periods (1930-1969; 1970-1989; 1990-2009). Since the period 1970-1989 contained fewer interactions between fewer species than the other periods, we did not take it into account in the analysis.

Finally, since the modularity of the selected networks (1930-1969; 1990-2009) and networks of intermediary periods (1940-1979; 1980-1999) was continuously declining (S2 Fig), this demonstrated the relevance of comparing the two large non-consecutive extreme periods, i.e. 1930-1969 and 1990-2009. They correspond to contrasted contexts related to major economic and land-use changes, i.e. respectively during and after the main intensification of agriculture [1].

S2 Fig. Selection of periods to compare. Boxplots of modularity per period of (a) 1000 random networks containing the same number of interactions or (b) the same number of interacting species (= nodes) as in the network whose dimensions are the weakest (i.e. (a) 541 interactions like in network of the period 1930-1969 and (b) and 99 bee species and 192 plants like in network of the period 1980-1999). Observed values of modularity are illustrated by red lines.

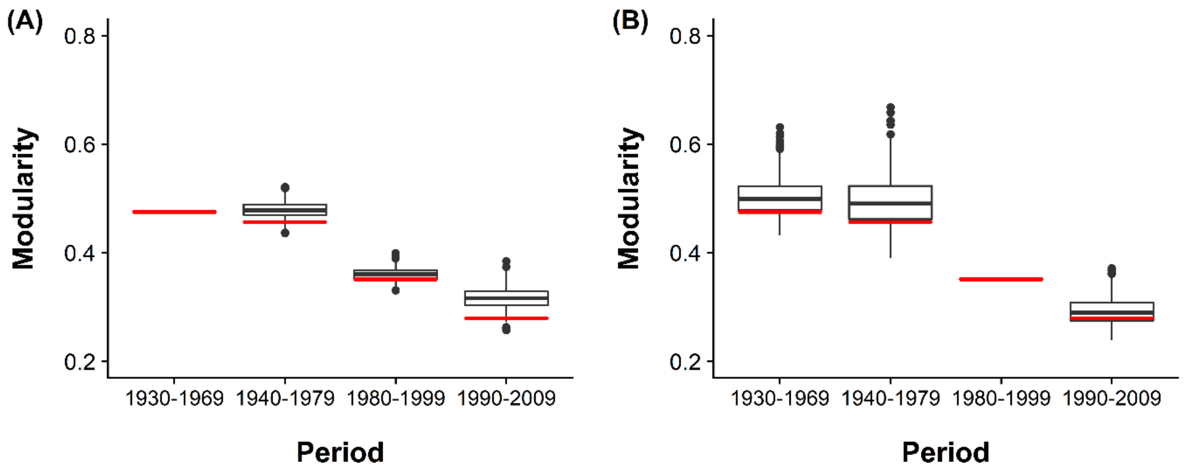

S3 Fig. Modularity of observed and resampled networks. The observed value of modularity of the period 1930-1969 is compared to modularity of resampled networks of the period 1990-2009 to have the same number of interactions as the period 1930-1969. Observed values of modularity are in red.

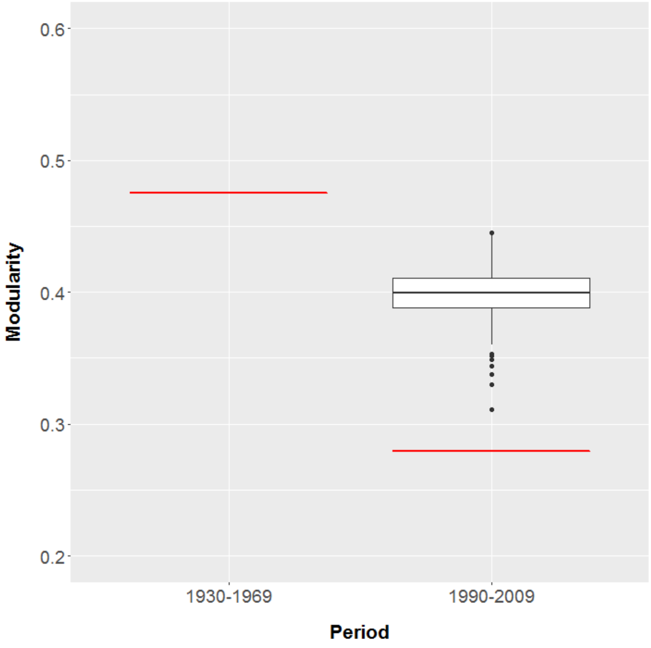

S4 Fig. Network structure per period. Structure and modularity (M) of the network (A) during the period 1930-1969 and (B) the period 1990-2009. The three main modules maintained in both periods are encircled. The key bee species of these three modules are indicated. The size of nodes representing bee species is proportional to the value of their participation coefficient (*c*) (the *z*-coefficient remaining constant overtime).

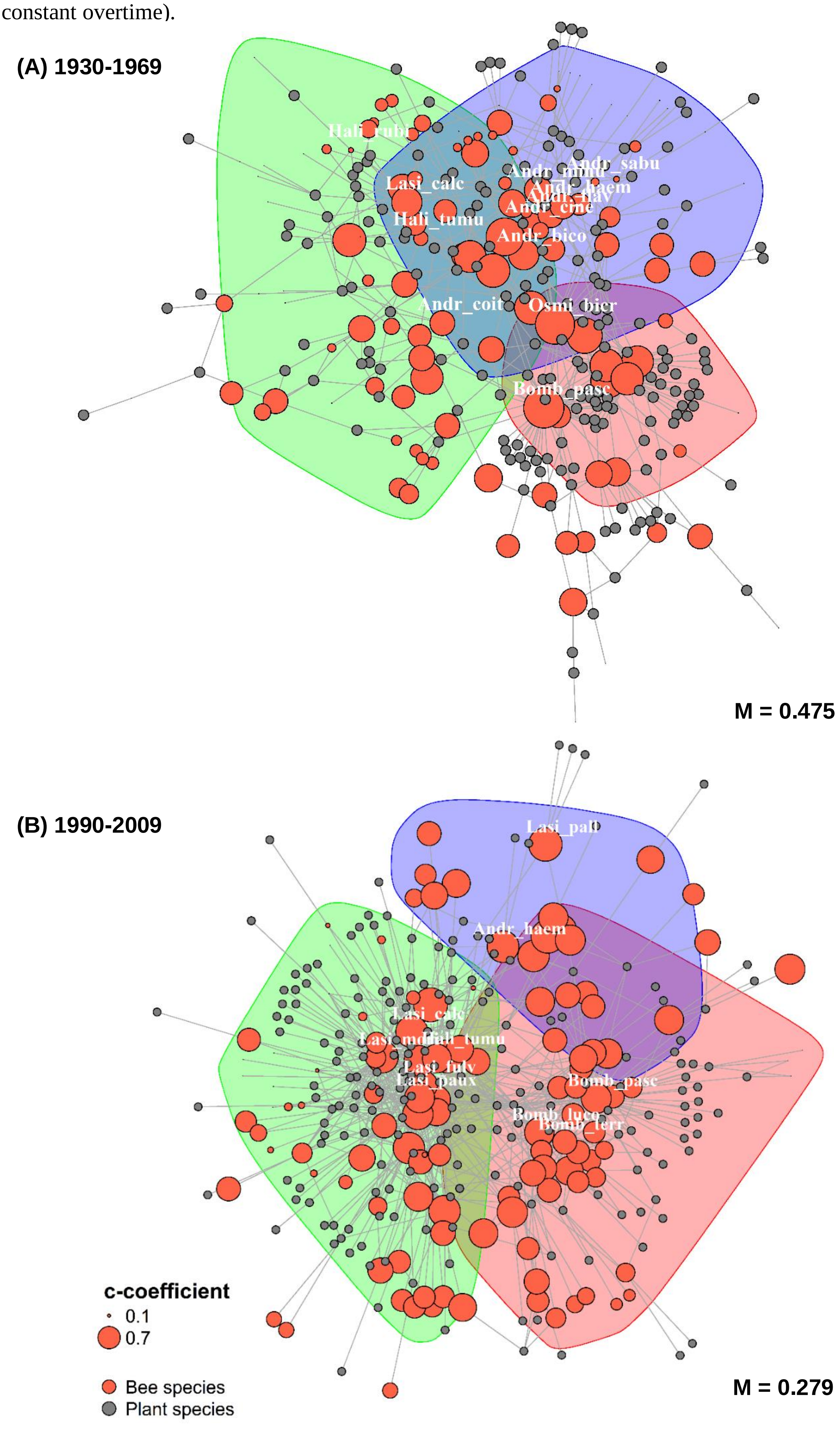

S5 Fig. Test of the homogeneity of the three main modules in terms of plant composition. Comparison of the ratio "number of species/number of genus" (in red) (following Elton [2]) of the three main modules to those of null models obtained by 1000 random permutations of the matrices plants*modules ("*independentswap*" algorithm, R package *picante*) for the period before 1970 (left) and the period after 1990 (right).

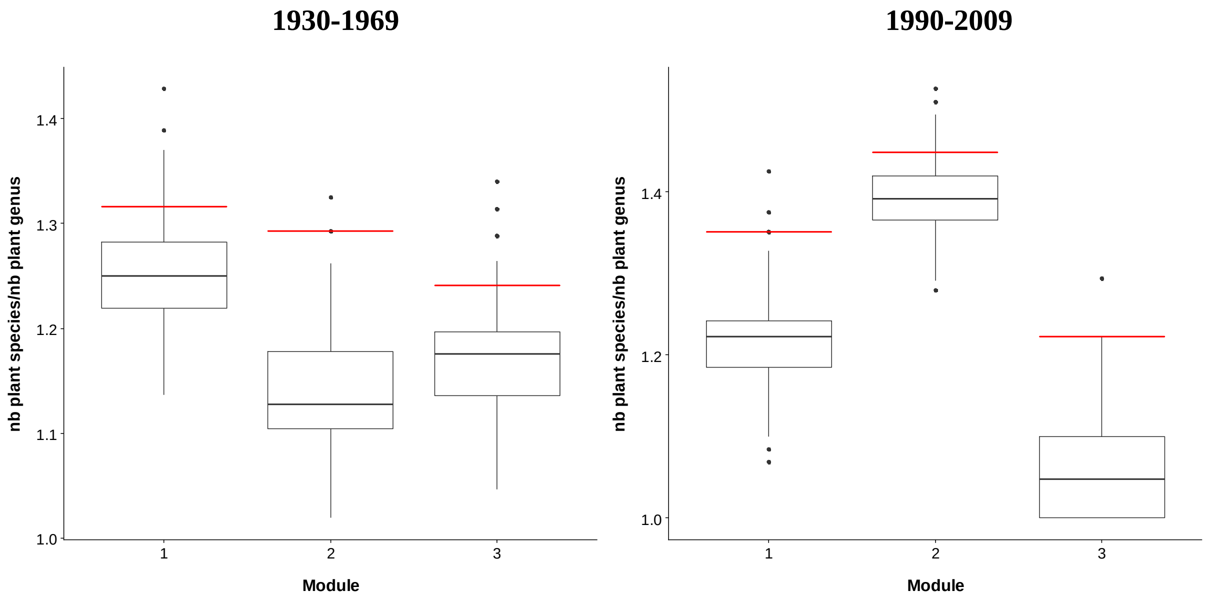

S6 Fig. Connectivity coefficient (*z*) of species observed within both periods in relation to their participation coefficient (*c*). Values of *cz*-coefficients (A) before 1970 (red) and (B) after 1990 (green). Species codes and full names are listed in S1 Table. Vertical and horizontal dashed lines represent 90% quantiles of null model coefficients and delimit groups of species with different topological roles in networks.

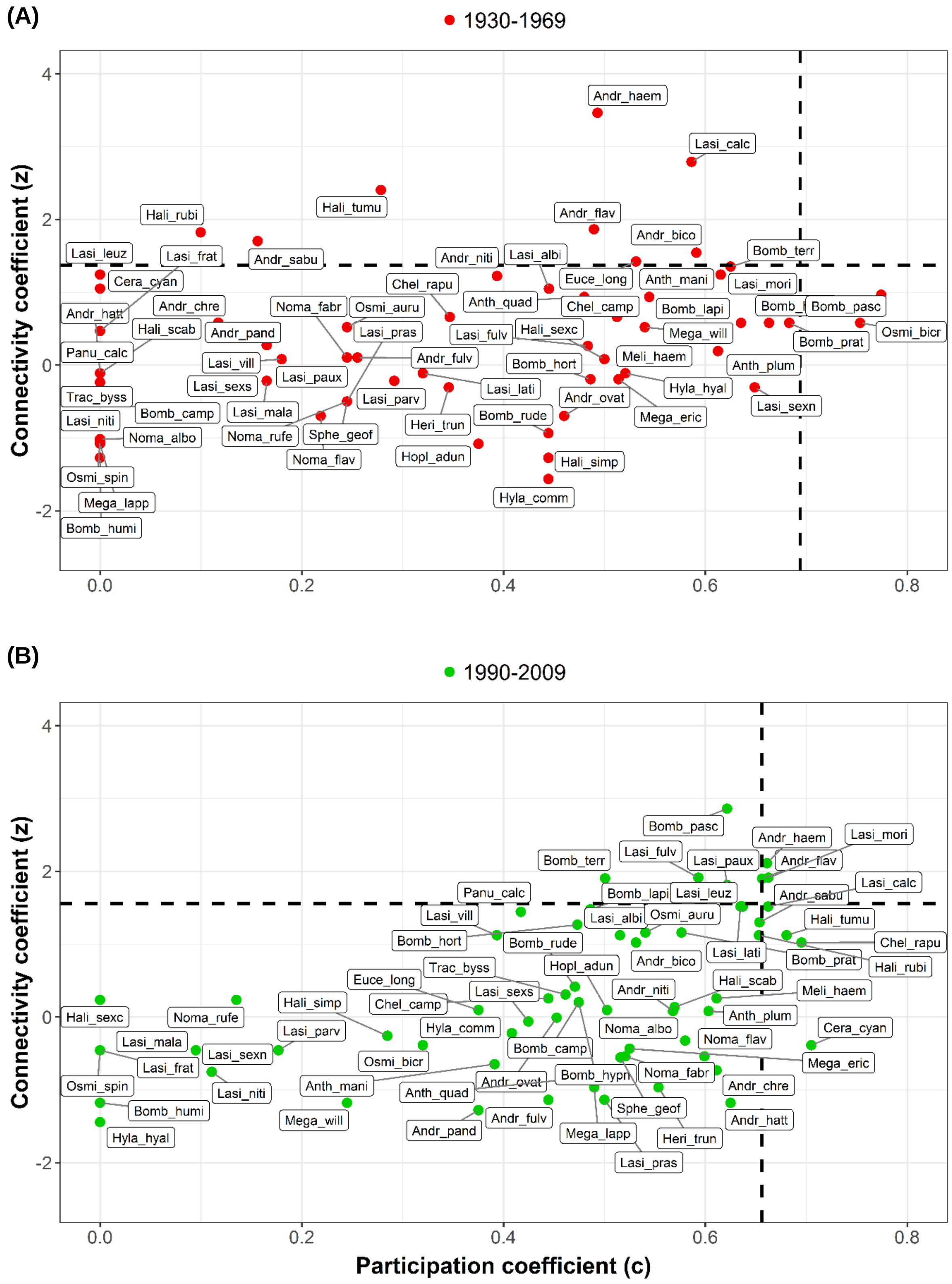

S7 Fig. Connectivity coefficient (z) of species observed on both periods in relation to their participation coefficient (c) before 1970 (red) related to the coefficients after 1990 (green).

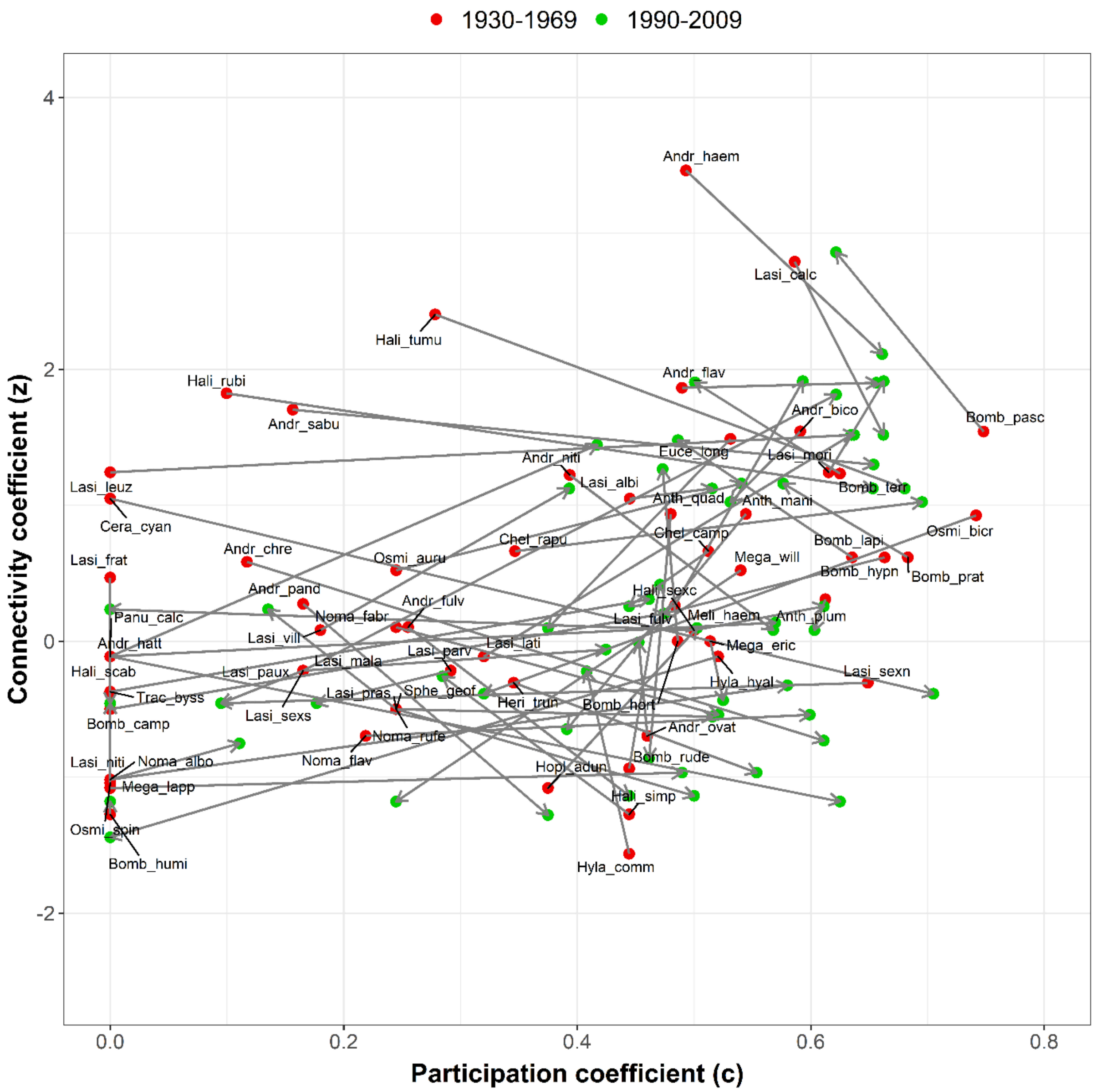

S8 Fig. Significance of correlation coefficient of Pearson between degree and *cz*-coefficients, all periods combined.

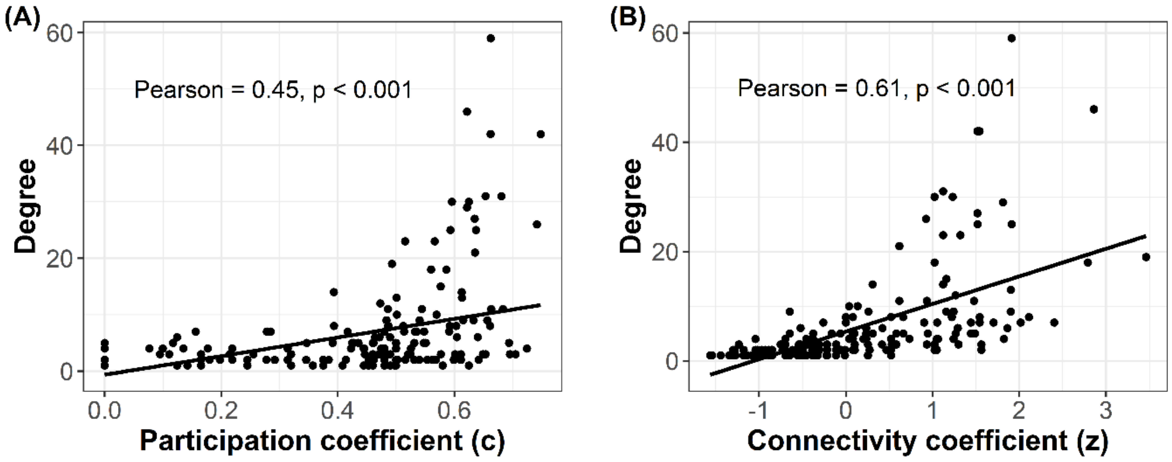

References

1. Kleijn D, Sutherland WJ. How effective are European agri-environment schemes in conserving and promoting biodiversity? J Appl Ecol. 2003;40: 947–969.

2. Elton C. Competition and the Structure of Ecological Communities. J Anim Ecol. 1946;15: 54–68.
