# Supplementary material for: Loss of pollinator specialization revealed by historical opportunistic data: Insights from network-based analysis
Source: PLoS One. 2020 Jul 13;15(7):e0235890. doi: 10.1371/journal.pone.0235890 (PMC7357768; doi:10.1371/journal.pone.0235890)
Supplement: S1 Table — (PDF) [file pone.0235890.s002.pdf]

S1 Table. Code, full name, family and degree of interacting plant and bee species per period (1930-1969 and 1990-2009).

	Species code	Species	Family	< 1970	>= 1990
Bee species	Andr_agil	*Andrena agilissima*	Andrenidae	1	0
	Andr_angu	*Andrena angustior*	Andrenidae	6	0
	Andr_apic	*Andrena apicata*	Andrenidae	1	0
	Andr_barb	*Andrena barbilabris*	Andrenidae	2	0
	Andr_bico	*Andrena bicolor*	Andrenidae	7	2
	Andr_chre	*Andrena chrysosceles*	Andrenidae	5	2
	Andr_cine	*Andrena cineraria*	Andrenidae	8	0
	Andr_coit	*Andrena coitana*	Andrenidae	7	0
	Andr_comb	*Andrena combinata*	Andrenidae	1	0
	Andr_curv	*Andrena curvungula*	Andrenidae	4	0
	Andr_fals	*Andrena falsifica*	Andrenidae	1	0
	Andr_flav	*Andrena flavipes*	Andrenidae	6	9
	Andr_flor	*Andrena florea*	Andrenidae	2	0
	Andr_fuca	*Andrena fucata*	Andrenidae	3	0
	Andr_fulv	*Andrena fulva*	Andrenidae	3	1
	Andr_fusc	*Andrena fuscipes*	Andrenidae	1	0
	Andr_grav	*Andrena gravida*	Andrenidae	4	0
	Andr_haem	*Andrena haemorrhoa*	Andrenidae	19	8
	Andr_hatt	*Andrena hattorfiana*	Andrenidae	1	1
	Andr_helv	*Andrena helvola*	Andrenidae	6	0
	Andr_labi	*Andrena labialis*	Andrenidae	2	0
	Andr_labt	*Andrena labiata*	Andrenidae	3	0
	Andr_lapp	*Andrena lapponica*	Andrenidae	3	0
	Andr_lath	*Andrena lathyri*	Andrenidae	3	0
	Andr_marg	*Andrena marginata*	Andrenidae	2	0
	Andr_minu	*Andrena minutula*	Andrenidae	7	0
	Andr_niga	*Andrena nigroaenea*	Andrenidae	5	0
	Andr_niti	*Andrena nitida*	Andrenidae	8	2
	Andr_ovat	*Andrena ovatula*	Andrenidae	3	1
	Andr_pand	*Andrena pandellei*	Andrenidae	2	2
	Andr_pili	*Andrena pilipes*	Andrenidae	3	0
	Andr_pote	*Andrena potentillae*	Andrenidae	1	0
	Andr_prox	*Andrena proxima*	Andrenidae	4	0
	Andr_rosa	*Andrena rosae*	Andrenidae	1	0
	Andr_rufi	*Andrena ruficrus*	Andrenidae	2	0
	Andr_sabu	*Andrena sabulosa*	Andrenidae	7	3
	Andr_sche	*Andrena schencki*	Andrenidae	3	0
	Andr_semi	*Andrena semilaevis*	Andrenidae	4	0
	Andr_simi	*Andrena similis*	Andrenidae	2	0
	Andr_stro	*Andrena strohmella*	Andrenidae	1	0
	Andr_subo	*Andrena subopaca*	Andrenidae	7	0
	Andr_syna	*Andrena synadelpha*	Andrenidae	1	0

	Andr_tibi	*Andrena tibialis*	Andrenidae	1	0
	Andr_vari	*Andrena varians*	Andrenidae	1	0
	Andr_wilk	*Andrena wilkella*	Andrenidae	3	0
	Anth_aest	*Anthophora aestivalis*	Apidae	2	0
	Anth_furc	*Anthophora furcata*	Apidae	1	0
	Anth_mani	*Anthidium manicatum*	Megachilidae	11	5
	Anth_oblo	*Anthidium oblongatum*	Megachilidae	0	3
	Anth_plum	*Anthophora plumipes*	Apidae	14	8
	Anth_punc	*Anthidium punctatum*	Megachilidae	0	1
	Anth_quad	*Anthophora quadrimaculata*	Apidae	3	2
	Anth_stri	*Anthidiellum strigatum*	Megachilidae	0	2
	Bomb_bohe	*Bombus bohemicus*	Apidae	0	7
	Bomb_camp	*Bombus campestris*	Apidae	1	4
	Bomb_cryp	*Bombus cryptarum*	Apidae	0	8
	Bomb_hort	*Bombus hortorum*	Apidae	8	12
	Bomb_humi	*Bombus humilis*	Apidae	1	2
	Bomb_hypn	*Bombus hypnorum*	Apidae	11	4
	Bomb_jone	*Bombus jonellus*	Apidae	0	1
	Bomb_lapi	*Bombus lapidarius*	Apidae	21	11
	Bomb_luco	*Bombus lucorum*	Apidae	0	13
	Bomb_magn	*Bombus magnus*	Apidae	0	1
	Bomb_norv	*Bombus norvegicus*	Apidae	0	2
	Bomb_pasc	*Bombus pascuorum*	Apidae	42	46
	Bomb_prat	*Bombus pratorum*	Apidae	11	15
	Bomb_rude	*Bombus ruderarius*	Apidae	1	3
	Bomb_rudt	*Bombus ruderatus*	Apidae	0	1
	Bomb_rupe	*Bombus rupestris*	Apidae	0	1
	Bomb_soro	*Bombus soroeensis*	Apidae	0	5
	Bomb_syli	*Bombus sylvestris*	Apidae	0	5
	Bomb_sylv	*Bombus sylvarum*	Apidae	0	2
	Bomb_terr	*Bombus terrestris*	Apidae	30	13
	Bomb_vest	*Bombus vestalis*	Apidae	0	4
	Cera_cyan	*Ceratina cyanea*	Apidae	2	3
	Chel_camp	*Chelostoma campanularum*	Megachilidae	8	1
	Chel_dist	*Chelostoma distinctum*	Megachilidae	1	0
	Chel_flor	*Chelostoma florisomne*	Megachilidae	1	0
	Chel_rapu	*Chelostoma rapunculi*	Megachilidae	5	3
	Coll_davi	*Colletes daviesanus*	Colletidae	3	2
	Coll_hede	*Colletes hederae*	Colletidae	0	1
	Coll_simi	*Colletes similis*	Colletidae	0	1
	Coll_succ	*Colletes succinctus*	Colletidae	0	1
	Dasy_hirt	*Dasypoda hirtipes*	Melittidae	0	4
	Dufo_dent	*Dufourea dentiventris*	Halictidae	2	0
	Dufo_minu	*Dufourea minuta*	Halictidae	2	0
	Euce_long	*Eucera longicornis*	Apidae	5	2

	Euce_nigr	*Eucera nigrescens*	Apidae	3	0
	Hali_comp	*Halictus compressus*	Halictidae	1	0
	Hali_conf	*Halictus confusus*	Halictidae	0	1
	Hali_macu	*Halictus maculatus*	Halictidae	0	18
	Hali_quad	*Halictus quadricinctus*	Halictidae	2	0
	Hali_rubi	*Halictus rubicundus*	Halictidae	4	31
	Hali_scab	*Halictus scabiosae*	Halictidae	1	10
	Hali_sexc	*Halictus sexcinctus*	Halictidae	4	5
	Hali_simp	*Halictus simplex*	Halictidae	1	7
	Hali_tumu	*Halictus tumulorum*	Halictidae	7	31
	Heri_trun	*Heriades truncorum*	Megachilidae	2	2
	Hopl_adun	*Hoplitis adunca*	Megachilidae	1	2
	Hopl_anth	*Hoplitis anthocopoides*	Megachilidae	1	0
	Hopl_clav	*Hoplitis claviventris*	Megachilidae	1	0
	Hopl_papa	*Hoplitis papaveris*	Megachilidae	2	0
	Hopl_ravo	*Hoplitis ravouxi*	Megachilidae	0	1
	Hyla_brev	*Hylaeus brevicornis*	Colletidae	0	1
	Hyla_comm	*Hylaeus communis*	Colletidae	1	1
	Hyla_conf	*Hylaeus confusus*	Colletidae	0	3
	Hyla_corn	*Hylaeus cornutus*	Colletidae	0	1
	Hyla_gred	*Hylaeus gredleri*	Colletidae	0	1
	Hyla_hyal	*Hylaeus hyalinatus*	Colletidae	3	1
	Hyla_punc	*Hylaeus punctulatissimus*	Colletidae	1	0
	Hyla_sign	*Hylaeus signatus*	Colletidae	0	1
	Lasi_albi	*Lasioglossum albipes*	Halictidae	4	23
	Lasi_calc	*Lasioglossum calceatum*	Halictidae	18	42
	Lasi_frat	*Lasioglossum fratellum*	Halictidae	2	1
	Lasi_fulv	*Lasioglossum fulvicorne*	Halictidae	9	25
	Lasi_inte	*Lasioglossum interruptum*	Halictidae	0	1
	Lasi_laev	*Lasioglossum laevigatum*	Halictidae	0	5
	Lasi_lati	*Lasioglossum laticeps*	Halictidae	3	27
	Lasi_latv	*Lasioglossum lativentre*	Halictidae	0	10
	Lasi_leuc	*Lasioglossum leucopus*	Halictidae	0	6
	Lasi_leuz	*Lasioglossum leucozonium*	Halictidae	4	25
	Lasi_line	*Lasioglossum lineare*	Halictidae	1	0
	Lasi_mala	*Lasioglossum malachurum*	Halictidae	1	3
	Lasi_mint	*Lasioglossum minutulum*	Halictidae	0	4
	Lasi_mori	*Lasioglossum morio*	Halictidae	9	59
	Lasi_niti	*Lasioglossum nitidiusculum*	Halictidae	2	3
	Lasi_nitu	*Lasioglossum nitidulum*	Halictidae	0	23
	Lasi_pall	*Lasioglossum pallens*	Halictidae	0	5
	Lasi_parv	*Lasioglossum parvulum*	Halictidae	2	4
	Lasi_paux	*Lasioglossum pauxillum*	Halictidae	3	29
	Lasi_pras	*Lasioglossum prasinum*	Halictidae	1	1
	Lasi_punc	*Lasioglossum punctatissimum*	Halictidae	0	6

	Lasi_pygm	*Lasioglossum pygmaeum*	Halictidae	0	2
	Lasi_quad	*Lasioglossum quadrinotatulum*	Halictidae	0	2
	Lasi_semi	*Lasioglossum semilucens*	Halictidae	0	1
	Lasi_sexn	*Lasioglossum sexnotatum*	Halictidae	3	3
	Lasi_sexs	*Lasioglossum sexstrigatum*	Halictidae	2	5
	Lasi_vill	*Lasioglossum villosulum*	Halictidae	2	14
	Lasi_zonu	*Lasioglossum zonulum*	Halictidae	0	30
	Macr_euro	*Macropis europaea*	Melittidae	1	1
	Macr_fulv	*Macropis fulvipes*	Melittidae	3	1
	Mega_alpi	*Megachile alpicola*	Megachilidae	1	0
	Mega_cent	*Megachile centuncularis*	Megachilidae	0	2
	Mega_eric	*Megachile ericetorum*	Megachilidae	7	3
	Mega_lago	*Megachile lagopoda*	Megachilidae	1	0
	Mega_lapp	*Megachile lapponica*	Megachilidae	1	1
	Mega_pyre	*Megachile pyrenaea*	Megachilidae	0	1
	Mega_vers	*Megachile versicolor*	Megachilidae	1	0
	Mega_will	*Megachile willughbiella*	Megachilidae	2	1
	Mele_albi	*Melecta albifrons*	Apidae	2	0
	Meli_haem	*Melitta haemorrhoidalis*	Melittidae	5	3
	Meli_lepo	*Melitta leporina*	Melittidae	2	0
	Meli_nigr	*Melitta nigricans*	Melittidae	0	1
	Meli_tric	*Melitta tricincta*	Melittidae	2	0
	Noma_albo	*Nomada alboguttata*	Apidae	1	1
	Noma_fabr	*Nomada fabriciana*	Apidae	2	3
	Noma_flao	*Nomada flavoguttata*	Apidae	1	0
	Noma_flav	*Nomada flava*	Apidae	4	2
	Noma_good	*Nomada goodeniana*	Apidae	1	0
	Noma_leuc	*Nomada leucophthalma*	Apidae	0	1
	Noma_mars	*Nomada marshamella*	Apidae	0	2
	Noma_rufe	*Nomada rufipes*	Apidae	1	4
	Noma_rufi	*Nomada ruficornis*	Apidae	1	0
	Noma_stig	*Nomada stigma*	Apidae	0	1
	Noma_stri	*Nomada striata*	Apidae	1	0
	Osmi_andr	*Osmia andrenoides*	Megachilidae	2	0
	Osmi_auru	*Osmia aurulenta*	Megachilidae	1	9
	Osmi_bico	*Osmia bicolor*	Megachilidae	0	9
	Osmi_bicr	*Osmia bicornis*	Megachilidae	26	1
	Osmi_caer	*Osmia caerulescens*	Megachilidae	0	1
	Osmi_corn	*Osmia cornuta*	Megachilidae	6	0
	Osmi_nive	*Osmia niveata*	Megachilidae	0	1
	Osmi_pili	*Osmia pilicornis*	Megachilidae	2	0
	Osmi_rufo	*Osmia rufohirta*	Megachilidae	2	0
	Osmi_spin	*Osmia spinulosa*	Megachilidae	2	1
	Osmi_xant	*Osmia xanthomelana*	Megachilidae	1	0
	Panu_bank	*Panurgus banksianus*	Andrenidae	0	2

	Panu_calc	*Panurgus calcaratus*	Andrenidae	1	7
	Roph_quin	*Rophites quinquespinosus*	Halictidae	0	1
	Sphe_albi	*Sphecodes albilabris*	Halictidae	0	5
	Sphe_cras	*Sphecodes crassus*	Halictidae	0	1
	Sphe_ephi	*Sphecodes ephippius*	Halictidae	0	5
	Sphe_ferr	*Sphecodes ferruginatus*	Halictidae	0	3
	Sphe_geof	*Sphecodes geoffrellus*	Halictidae	1	2
	Sphe_gibb	*Sphecodes gibbus*	Halictidae	2	1
	Sphe_hyal	*Sphecodes hyalinatus*	Halictidae	0	3
	Sphe_moni	*Sphecodes monilicornis*	Halictidae	0	2
	Sphe_pell	*Sphecodes pellucidus*	Halictidae	0	1
	Sphe_rufv	*Sphecodes rufiventris*	Halictidae	0	1
	Trac_byss	*Trachusa byssina*	Megachilidae	1	5
Plant species	Acer_camp	*Acer campestre*	Aceraceae	8	0
	Acer_pseu	*Acer pseudoplatanus*	Aceraceae	7	1
	Achi_mill	*Achillea millefolium*	Asteraceae	1	8
	Acin_arve	*Acinos arvensis*	Lamiaceae	0	1
	Aego_poda	*Aegopodium podagraria*	Apiaceae	4	0
	Ajug_rept	*Ajuga reptans*	Lamiaceae	0	1
	Alce_rose	*Alcea rosea*	Malvaceae	1	0
	Alli_cepa	*Allium cepa*	Amaryllidaceae	1	0
	Alli_peti	*Alliaria petiolata*	Brassicaceae	5	1
	Alli_spha	*Allium sphaerocephalon*	Amaryllidaceae	0	8
	Alli_ursi	*Allium ursinum*	Amaryllidaceae	1	1
	Alys_saxa	*Alyssum saxatile*	Brassicaceae	1	0
	Anch_offi	*Anchusa officinalis*	Boraginaceae	7	0
	Andr_poli	*Andromeda polifolia*	Ericaceae	0	2
	Anem_coro	*Anemone coronaria*	Ranunculaceae	1	0
	Anem_nemo	*Anemone nemorosa*	Ranunculaceae	1	0
	Ange_arch	*Angelica archangelica*	Apiaceae	1	0
	Ange_sylv	*Angelica sylvestris*	Apiaceae	1	4
	Anth_lili	*Anthericum liliago*	Asparagaceae	0	2
	Anth_sylv	*Anthriscus sylvestris*	Apiaceae	0	2
	Anth_vuln	*Anthyllis vulneraria*	Fabaceae	1	0
	Anti_maju	*Antirrhinum majus*	Plantaginaceae	1	0
	Aqui_vulg	*Aquilegia vulgaris*	Ranunculaceae	3	0
	Arab_alpi	*Arabis alpina*	Brassicaceae	2	0
	Arab_hirs	*Arabis hirsuta*	Brassicaceae	0	2
	Arme_mari	*Armeria maritima*	Plumbaginaceae	3	0
	Aste_lino	*Aster linosyris*	Asteraceae	0	7
	Astr_glyc	*Astragalus glycyphyllos*	Fabaceae	0	1
	Atro_bell	*Atropa bella-donna*	Solanaceae	1	1
	Aubr_delt	*Aubrieta deltoidea*	Brassicaceae	1	0
	Ball_nigr	*Ballota nigra*	Lamiaceae	1	0
	Barb_vulg	*Barbarea vulgaris*	Brassicaceae	1	0

	Bell_pere	*Bellis perennis*	Asteraceae	6	4
	Bert_inca	*Berteroa incana*	Brassicaceae	2	6
	Bide_cern	*Bidens cernua*	Asteraceae	0	1
	Bora_offi	*Borago officinalis*	Boraginaceae	0	2
	Bras_oler	*Brassica oleracea*	Brassicaceae	0	1
	Bryo_dioi	*Bryonia dioica*	Cucurbitaceae	6	1
	Budd_davi	*Buddleja davidii*	Scrophulariaceae	1	1
	Bupl_falc	*Bupleurum falcatum*	Apiaceae	0	2
	Cale_offi	*Calendula officinalis*	Asteraceae	1	0
	Call_vulg	*Calluna vulgaris*	Ericaceae	2	13
	Caly_sepi	*Calystegia sepium*	Convolvulaceae	0	3
	Camp_patu	*Campanula patula*	Campanulaceae	1	0
	Camp_pers	*Campanula persicifolia*	Campanulaceae	3	0
	Camp_rapn	*Campanula rapunculus*	Campanulaceae	5	3
	Camp_rapu	*Campanula rapunculoides*	Campanulaceae	2	0
	Camp_rotu	*Campanula rotundifolia*	Campanulaceae	9	6
	Camp_trac	*Campanula trachelium*	Campanulaceae	4	1
	Caps_burs	*Capsella bursa-pastoris*	Brassicaceae	2	0
	Card_cris	*Carduus crispus*	Asteraceae	4	6
	Card_hirs	*Cardamine hirsuta*	Brassicaceae	0	3
	Card_nuta	*Carduus nutans*	Asteraceae	0	2
	Card_prat	*Cardamine pratensis*	Brassicaceae	1	1
	Carp_betu	*Carpinus betulus*	Betulaceae	1	0
	Cent_cyan	*Centaurea cyanus*	Asteraceae	1	1
	Cent_jace	*Centaurea jacea*	Asteraceae	8	26
	Cent_scab	*Centaurea scabiosa*	Asteraceae	2	2
	Cera_font	*Cerastium fontanum*	Caryophyllaceae	0	1
	Cera_glom	*Cerastium glomeratum*	Caryophyllaceae	1	0
	Chae_temu	*Chaerophyllum temulum*	Apiaceae	1	0
	Cirs_arve	*Cirsium arvense*	Asteraceae	2	15
	Cirs_palu	*Cirsium palustre*	Asteraceae	0	6
	Cirs_vulg	*Cirsium vulgare*	Asteraceae	2	5
	Clar_ungu	*Clarkia unguiculata*	Onagraceae	3	0
	Clem_vita	*Clematis vitalba*	Ranunculaceae	0	5
	Clin_vulg	*Clinopodium vulgare*	Lamiaceae	0	1
	Conv_arve	*Convolvulus arvensis*	Convolvulaceae	1	1
	Cony_cana	*Conyza canadensis*	Asteraceae	0	1
	Corn_mas	*Cornus mas*	Cornaceae	2	0
	Corn_sang	*Cornus sanguinea*	Cornaceae	5	5
	Cory_soli	*Corydalis solida*	Papaveraceae	2	1
	Cosm_bipi	*Cosmos bipinnatus*	Asteraceae	2	1
	Coto_simo	*Cotoneaster simonsii*	Rosaceae	2	0
	Crat_mono	*Crataegus monogyna*	Rosaceae	0	9
	Crep_bien	*Crepis biennis*	Asteraceae	0	4
	Crep_capi	*Crepis capillaris*	Asteraceae	4	5

	Cruc_laev	*Cruciata laevipes*	Rubiaceae	1	0
	Cymb_mura	*Cymbalaria muralis*	Plantaginaceae	1	0
	Cyti_scop	*Cytisus scoparius*	Fabaceae	3	1
	Dauc_caro	*Daucus carota*	Apiaceae	7	6
	Dian_barb	*Dianthus barbatus*	Caryophyllaceae	1	0
	Dian_cary	*Dianthus caryophyllus*	Caryophyllaceae	1	0
	Dice_spec	*Dicentra spectabilis*	Papaveraceae	1	0
	Digi_lute	*Digitalis lutea*	Plantaginaceae	1	4
	Digi_purp	*Digitalis purpurea*	Plantaginaceae	5	0
	Dips_full	*Dipsacus fullonum*	Caprifoliaceae	0	1
	Dips_pilo	*Dipsacus pilosus*	Caprifoliaceae	0	1
	Drab_mura	*Draba muralis*	Brassicaceae	0	1
	Echi_ritr	*Echinops ritro*	Asteraceae	0	1
	Echi_spha	*Echinops sphaerocephalus*	Asteraceae	3	0
	Echi_vulg	*Echium vulgare*	Boraginaceae	4	21
	Epil_angu	*Epilobium angustifolium*	Onagraceae	3	14
	Epil_hirs	*Epilobium hirsutum*	Onagraceae	1	4
	Epil_parv	*Epilobium parviflorum*	Onagraceae	0	2
	Eric_carn	*Erica carnea*	Ericaceae	1	0
	Eryn_plan	*Eryngium planum*	Apiaceae	0	2
	Erys_chei	*Erysimum cheiri*	Brassicaceae	3	0
	Eupa_cann	*Eupatorium cannabinum*	Asteraceae	0	9
	Euph_cypa	*Euphorbia cyparissias*	Euphorbiaceae	0	3
	Euph_esul	*Euphorbia esula*	Euphorbiaceae	1	0
	Euph_lath	*Euphorbia lathyris*	Euphorbiaceae	0	1
	Foen_vulg	*Foeniculum vulgare*	Apiaceae	0	1
	Frag_vesc	*Fragaria vesca*	Rosaceae	0	7
	Fran_alnu	*Frangula alnus*	Rhamnaceae	5	0
	Gala_niva	*Galanthus nivalis*	Amaryllidaceae	1	0
	Gale_offi	*Galega officinalis*	Fabaceae	0	1
	Gale_tetr	*Galeopsis tetrahit*	Lamiaceae	1	0
	Gali_aren	*Galium arenarium*	Rubiaceae	0	1
	Geni_angl	*Genista anglica*	Fabaceae	1	0
	Geni_pilo	*Genista pilosa*	Fabaceae	1	0
	Geni_tinc	*Genista tinctoria*	Fabaceae	0	1
	Gera_diss	*Geranium dissectum*	Geraniaceae	0	1
	Gera_moll	*Geranium molle*	Geraniaceae	0	2
	Gera_pusi	*Geranium pusillum*	Geraniaceae	2	0
	Gera_pyre	*Geranium pyrenaicum*	Geraniaceae	1	6
	Gera_robe	*Geranium robertianum*	Geraniaceae	3	2
	Gera_sang	*Geranium sanguineum*	Geraniaceae	3	0
	Geum_riva	*Geum rivale*	Rosaceae	0	1
	Glec_hede	*Glechoma hederacea*	Lamiaceae	7	2
	Glob_bisn	*Globularia bisnagarica*	Plantaginaceae	0	3
	Hede_heli	*Hedera helix*	Araliaceae	0	2

	Hele_autu	*Helenium autumnale*	Asteraceae	1	0
	Heli_apen	*Helianthemum apenninum*	Cistaceae	0	1
	Heli_numm	*Helianthemum nummularium*	Cistaceae	0	2
	Hell_viri	*Helleborus viridis*	Ranunculaceae	2	0
	Hera_spho	*Heracleum sphondylium*	Apiaceae	5	8
	Hibi_syri	*Hibiscus syriacus*	Malvaceae	1	0
	Hier_bauh	*Hieracium bauhinii*	Asteraceae	0	1
	Hier_flag	*Hieracium flagellare*	Asteraceae	0	3
	Hier_lach	*Hieracium lachenalii*	Asteraceae	0	1
	Hier_laev	*Hieracium laevigatum*	Asteraceae	3	0
	Hier_muro	*Hieracium murorum*	Asteraceae	0	2
	Hier_pilo	*Hieracium pilosella*	Asteraceae	0	11
	Hier_saba	*Hieracium sabaudum*	Asteraceae	0	1
	Hier_umbe	*Hieracium umbellatum*	Asteraceae	0	4
	Hipp_como	*Hippocrepis comosa*	Fabaceae	3	3
	Hype_macu	*Hypericum maculatum*	Hypericaceae	0	1
	Hype_perf	*Hypericum perforatum*	Hypericaceae	2	6
	Hype_perl	*Hypericum perfoliatum*	Hypericaceae	0	1
	Hypo_radi	*Hypochaeris radicata*	Asteraceae	8	7
	Hyss_offi	*Hyssopus officinalis*	Lamiaceae	4	0
	Impa_glan	*Impatiens glandulifera*	Balsaminaceae	0	5
	Inul_cony	*Inula conyzae*	Asteraceae	0	3
	Inul_hele	*Inula helenium*	Asteraceae	0	1
	Inul_sali	*Inula salicina*	Asteraceae	1	7
	Iris_germ	*Iris germanica*	Iridaceae	1	0
	Iris_pseu	*Iris pseudacorus*	Iridaceae	1	0
	Jasi_mont	*Jasione montana*	Campanulaceae	0	1
	Knau_arve	*Knautia arvensis*	Caprifoliaceae	1	12
	Lact_pere	*Lactuca perennis*	Asteraceae	0	1
	Lami_albu	*Lamium album*	Lamiaceae	6	13
	Lami_gale	*Lamium galeobdolon*	Lamiaceae	1	0
	Lami_hybr	*Lamium hybridum*	Lamiaceae	1	0
	Lami_macu	*Lamium maculatum*	Lamiaceae	2	0
	Lami_orva	*Lamium orvala*	Lamiaceae	0	1
	Lami_purp	*Lamium purpureum*	Lamiaceae	1	2
	Laps_comm	*Lapsana communis*	Asteraceae	0	1
	Lath_lati	*Lathyrus latifolius*	Fabaceae	1	0
	Lath_lini	*Lathyrus linifolius*	Fabaceae	3	0
	Lath_prat	*Lathyrus pratensis*	Fabaceae	3	0
	Lath_sylv	*Lathyrus sylvestris*	Fabaceae	4	0
	Lava_angu	*Lavandula angustifolia*	Lamiaceae	0	6
	Lava_x.in	*Lavandula x.intermedia*	Lamiaceae	0	3
	Leon_autu	*Leontodon autumnalis*	Asteraceae	2	5
	Leon_hisp	*Leontodon hispidus*	Asteraceae	0	2
	Lepi_camp	*Lepidium campestre*	Brassicaceae	0	2

	Leuc_vulg	*Leucanthemum vulgare*	Asteraceae	1	10
	Ligu_vulg	*Ligustrum vulgare*	Oleaceae	0	1
	Limo_vulg	*Limonium vulgare*	Plumbaginaceae	1	0
	Lina_purp	*Linaria purpurea*	Plantaginaceae	1	0
	Lina_repe	*Linaria repens*	Plantaginaceae	0	2
	Lina_vulg	*Linaria vulgaris*	Plantaginaceae	2	5
	Lith_offi	*Lithospermum officinale*	Boraginaceae	0	4
	Loni_peri	*Lonicera periclymenum*	Caprifoliaceae	4	0
	Lotu_corn	*Lotus corniculatus*	Fabaceae	7	14
	Lotu_pedu	*Lotus pedunculatus*	Fabaceae	1	1
	Lupi_lute	*Lupinus luteus*	Fabaceae	2	0
	Lyco_euro	*Lycopus europaeus*	Lamiaceae	0	1
	Lysi_punc	*Lysimachia punctata*	Primulaceae	0	2
	Lysi_vulg	*Lysimachia vulgaris*	Primulaceae	2	3
	Lyth_sali	*Lythrum salicaria*	Lythraceae	0	5
	Maho_aqui	*Mahonia aquifolium*	Berberidaceae	4	0
	Malu_sylv	*Malus sylvestris*	Rosaceae	2	0
	Malv_mosc	*Malva moschata*	Malvaceae	3	7
	Malv_sylv	*Malva sylvestris*	Malvaceae	6	1
	Marr_vulg	*Marrubium vulgare*	Lamiaceae	4	0
	Matr_cham	*Matricaria chamomilla*	Asteraceae	1	0
	Matr_mari	*Matricaria maritima*	Asteraceae	0	5
	Medi_sati	*Medicago sativa*	Fabaceae	2	0
	Meli_albu	*Melilotus albus*	Fabaceae	0	2
	Meli_alti	*Melilotus altissimus*	Fabaceae	0	2
	Meli_offi	*Melilotus officinalis*	Fabaceae	1	0
	Ment_aqua	*Mentha aquatica*	Lamiaceae	0	3
	Miso_oron	*Misopates orontium*	Plantaginaceae	1	0
	Myos_aqua	*Myosoton aquaticum*	Caryophyllaceae	1	1
	Nepe_cata	*Nepeta cataria*	Lamiaceae	2	0
	Odon_vern	*Odontites vernus*	Orobanchaceae	1	0
	Oeno_defl	*Oenothera deflexa*	Onagraceae	0	1
	Onob_vici	*Onobrychis viciifolia*	Fabaceae	0	2
	Onon_repe	*Ononis repens*	Fabaceae	0	4
	Onon_spin	*Ononis spinosa*	Fabaceae	1	0
	Orig_vulg	*Origanum vulgare*	Lamiaceae	2	15
	Papa_orie	*Papaver orientale*	Papaveraceae	2	0
	Papa_rhoe	*Papaver rhoeas*	Papaveraceae	0	2
	Past_sati	*Pastinaca sativa*	Apiaceae	0	1
	Pers_bist	*Persicaria bistorta*	Polygonaceae	1	1
	Pers_macu	*Persicaria maculosa*	Polygonaceae	0	1
	Phac_tana	*Phacelia tanacetifolia*	Boraginaceae	3	2
	Phas_vulg	*Phaseolus vulgaris*	Fabaceae	3	0
	Picr_hier	*Picris hieracioides*	Asteraceae	3	19
	Pimp_saxi	*Pimpinella saxifraga*	Apiaceae	1	0

	Poly_vulg	*Polygala vulgaris*	Polygalaceae	0	1
	Pote_erec	*Potentilla erecta*	Rosaceae	3	0
	Pote_neum	*Potentilla neumanniana*	Rosaceae	7	14
	Pote_ster	*Potentilla sterilis*	Rosaceae	5	0
	Prim_elat	*Primula elatior*	Primulaceae	1	0
	Prim_veri	*Primula veris*	Primulaceae	0	2
	Prim_vulg	*Primula vulgaris*	Primulaceae	1	0
	Prun_aviu	*Prunus avium*	Rosaceae	3	1
	Prun_cers	*Prunus cerasus*	Rosaceae	3	0
	Prun_dome	*Prunus domestica*	Rosaceae	1	1
	Prun_spin	*Prunus spinosa*	Rosaceae	5	6
	Pulm_moll	*Pulmonaria mollis*	Boraginaceae	2	0
	Pulm_offi	*Pulmonaria officinalis*	Boraginaceae	2	0
	Pyru_comm	*Pyrus communis*	Rosaceae	3	0
	Quer_pube	*Quercus pubescens*	Fagaceae	1	0
	Ranu_acri	*Ranunculus acris*	Ranunculaceae	5	1
	Ranu_bulb	*Ranunculus bulbosus*	Ranunculaceae	0	7
	Ranu_fica	*Ranunculus ficaria*	Ranunculaceae	9	2
	Ranu_repe	*Ranunculus repens*	Ranunculaceae	1	5
	Rese_lute	*Reseda lutea*	Resedaceae	6	9
	Rham_cath	*Rhamnus cathartica*	Rhamnaceae	1	1
	Ribe_rubr	*Ribes rubrum*	Grossulariaceae	1	0
	Rosa_cani	*Rosa canina*	Rosaceae	3	3
	Rubu_idae	*Rubus idaeus*	Rosaceae	4	2
	Rudb_laci	*Rudbeckia laciniata*	Asteraceae	3	0
	Sali_alba	*Salix alba*	Salicaceae	1	0
	Sali_auri	*Salix aurita*	Salicaceae	3	1
	Sali_capr	*Salix caprea*	Salicaceae	15	1
	Sali_purp	*Salix purpurea*	Salicaceae	0	1
	Salv_offi	*Salvia officinalis*	Lamiaceae	5	0
	Salv_verb	*Salvia verbenaca*	Lamiaceae	2	0
	Salv_vert	*Salvia verticillata*	Lamiaceae	1	0
	Samb_nigr	*Sambucus nigra*	Adoxaceae	1	0
	Sapo_offi	*Saponaria officinalis*	Caryophyllaceae	1	0
	Saxi_gran	*Saxifraga granulata*	Saxifragaceae	0	1
	Saxi_trid	*Saxifraga tridactylites*	Saxifragaceae	0	1
	Scab_colu	*Scabiosa columbaria*	Caprifoliaceae	6	4
	Scro_auri	*Scrophularia auriculata*	Scrophulariaceae	0	1
	Scro_nodo	*Scrophularia nodosa*	Scrophulariaceae	2	0
	Sedu_acre	*Sedum acre*	Crassulaceae	3	1
	Sedu_albu	*Sedum album*	Crassulaceae	0	1
	Sedu_rupe	*Sedum rupestre*	Crassulaceae	3	6
	Sedu_spur	*Sedum spurium*	Crassulaceae	3	0
	Sedu_tele	*Sedum telephium*	Crassulaceae	2	0
	Semp_tect	*Sempervivum tectorum*	Crassulaceae	1	0

	Sene_eruc	*Senecio erucifolius*	Asteraceae	0	3
	Sene_herc	*Senecio hercynicus*	Asteraceae	2	0
	Sene_inae	*Senecio inaequidens*	Asteraceae	0	4
	Sene_jaco	*Senecio jacobaea*	Asteraceae	7	15
	Sene_ovat	*Senecio ovatus*	Asteraceae	0	1
	Sese_liba	*Seseli libanotis*	Apiaceae	0	20
	Sila_sila	*Silaum silaus*	Apiaceae	0	2
	Sile_vulg	*Silene vulgaris*	Caryophyllaceae	1	0
	Sina_arve	*Sinapis arvensis*	Brassicaceae	0	2
	Sisy_alti	*Sisymbrium altissimum*	Brassicaceae	5	0
	Sisy_offi	*Sisymbrium officinale*	Brassicaceae	1	0
	Sola_dulc	*Solanum dulcamara*	Solanaceae	0	1
	Soli_cana	*Solidago canadensis*	Asteraceae	5	4
	Soli_giga	*Solidago gigantea*	Asteraceae	0	6
	Soli_virg	*Solidago virgaurea*	Asteraceae	2	18
	Sonc_arve	*Sonchus arvensis*	Asteraceae	0	9
	Sonc_aspe	*Sonchus asper*	Asteraceae	0	1
	Sorb_aucu	*Sorbus aucuparia*	Rosaceae	4	10
	Stac_offi	*Stachys officinalis*	Lamiaceae	3	7
	Stac_palu	*Stachys palustris*	Lamiaceae	0	1
	Stac_sylv	*Stachys sylvatica*	Lamiaceae	2	1
	Stel_holo	*Stellaria holostea*	Caryophyllaceae	1	0
	Stel_medi	*Stellaria media*	Caryophyllaceae	2	0
	Succ_prat	*Succisa pratensis*	Caprifoliaceae	3	4
	Symp_offi	*Symphytum officinale*	Boraginaceae	2	1
	Tana_vulg	*Tanacetum vulgare*	Asteraceae	1	8
	Tara_offi	*Taraxacum officinale*	Asteraceae	0	2
	Teuc_botr	*Teucrium botrys*	Lamiaceae	0	1
	Teuc_cham	*Teucrium chamaedrys*	Lamiaceae	0	11
	Teuc_scor	*Teucrium scorodonia*	Lamiaceae	5	2
	Thla_perf	*Thlaspi perfoliatum*	Brassicaceae	0	2
	Thym_prae	*Thymus praecox*	Lamiaceae	0	9
	Thym_pule	*Thymus pulegioides*	Lamiaceae	0	12
	Thym_serp	*Thymus serpyllum*	Lamiaceae	2	3
	Tili_plat	*Tilia platyphyllos*	Malvaceae	1	0
	Tori_japo	*Torilis japonica*	Apiaceae	0	1
	Trif_arve	*Trifolium arvense*	Fabaceae	0	1
	Trif_inca	*Trifolium incarnatum*	Fabaceae	0	4
	Trif_prat	*Trifolium pratense*	Fabaceae	3	8
	Trif_repe	*Trifolium repens*	Fabaceae	6	3
	Tuss_farf	*Tussilago farfara*	Asteraceae	11	1
	Ulex_euro	*Ulex europaeus*	Fabaceae	0	4
	Vacc_myrt	*Vaccinium myrtillus*	Ericaceae	1	2
	Vacc_ulig	*Vaccinium uliginosum*	Ericaceae	0	1
	Vale_offi	*Valeriana officinalis*	Caprifoliaceae	1	0

	Vale_repe	*Valeriana repens*	Caprifoliaceae	0	1
	Verb_thap	*Verbascum thapsus*	Scrophulariaceae	2	0
	Vero_cham	*Veronica chamaedrys*	Plantaginaceae	5	3
	Vero_offi	*Veronica officinalis*	Plantaginaceae	0	1
	Vero_spic	*Veronica spicata*	Plantaginaceae	6	1
	Vibu_opul	*Viburnum opulus*	Adoxaceae	1	1
	Vici_crac	*Vicia cracca*	Fabaceae	2	4
	Vici_hirs	*Vicia hirsuta*	Fabaceae	0	1
	Vici_sati	*Vicia sativa*	Fabaceae	2	2
	Vici_sepi	*Vicia sepium*	Fabaceae	4	0
	Vici_tetr	*Vicia tetrasperma*	Fabaceae	0	1
	Viol_odor	*Viola odorata*	Violaceae	1	0
	Viol_reic	*Viola reichenbachiana*	Violaceae	1	0
	Viol_tric	*Viola tricolor*	Violaceae	1	0
