# Supplementary material for: Loss of pollinator specialization revealed by historical opportunistic data: Insights from network-based analysis
Source: PLoS One. 2020 Jul 13;15(7):e0235890. doi: 10.1371/journal.pone.0235890 (PMC7357768; doi:10.1371/journal.pone.0235890)
Supplement: S2 Table — Module hubs has higher z-values and lower c, connectors has higher c-values but lower z and network hubs has both higher c and z. α = species that disappeared from the network after 1990; β = species that became peripheral after 1990; γ = peripheral species that became key species after 1990; δ = species that appeared in the network after 1990 as a key species; underlined = species that had a key role during both periods; in bold = species that kept their key role when we used the 95% quantiles of cz-coefficients of null models). Plant species with which they interacted the most before 1970 and after 1990 were the 10 species that have the maximum degree with key species. (PDF) [file pone.0235890.s003.pdf]

**S2 Table. Key bee species identified by comparing their *cz*-coefficients with thresholds corresponding to the 90% quantiles of *cz*-coefficients of null models.** Module hubs has higher *z*-values and lower *c*, connectors has higher *c*-values but lower *z* and network hubs has both higher *c* and *z*. $^{\alpha}$ = species that disappeared from the network after 1990; $^{\beta}$ = species that became peripheral after 1990; $^{\gamma}$ = peripheral species that became key species after 1990; $^{\delta}$ = species that appeared in the network after 1990 as a key species; underlined = species that had a key role during both periods; in bold = species that kept their key role when we used the 95% quantiles of *cz*-coefficients of null models). Plant species with which they interacted the most before 1970 and after 1990 were the 10 species that have the maximum degree of interaction with key species.

| | **1930-1969** | **1990-2009** |
|---|---|---|
| **Module hubs** | *Andrena bicolor*$^{\beta}$, *A. cineraria*$^{\alpha}$, ***A. coitana***$^{\alpha}$, *A. flavipes*, ***A. haemorrhoa***, *A. labiata*$^{\alpha}$, *Andrena lathyri*$^{\alpha}$, *A. minutula*$^{\alpha}$, *A. sabulosa*$^{\beta}$, *Eucera longicornis*$^{\beta}$, *Halictus rubicundus*$^{\beta}$, ***H. tumulorum***, ***Lasioglossum calceatum*** and *Osmia rufohirta*$^{\alpha}$ | *Bombus lucorum*$^{\delta}$, ***B. pascuorum***, *B. terrestris*$^{\gamma}$, *Lasioglossum fulvicorne*$^{\gamma}$, *L. pauxillum*$^{\gamma}$ |
| **Network hubs** | | *Andrena flavipes*, ***A. haemorrhoa*****, *Lasioglossum morio*$^{\gamma}$ |
| **Connectors** | *Andrena proxima*$^{\alpha,}$ ***Bombus pascuorum*** and ***Osmia bicornis***$^{\beta}$ | ***Ceratina cyanea***$^{\gamma}$, ***Chelostoma rapunculi***$^{\gamma}$, *Halictus tumulorum*, *Lasioglossum calceatum*, ***L. pallens***$^{\delta}$ |
| **Plant species** (degree) | *Salix caprea* (5), *Tussilago farfara* (5), *Glechoma hederacea* (4), *Prunus spinosa (4)*, *Bryonia dioica* (3), *Heracleum sphondylium* (3), *Hypochaeris radicata* (3), *Lotus corniculatus* (3), *Prunus cerasus* (3) and *Ranunculus acris* (3) | *Centaurea jacea* (6), *Echium vulgare* (6), *Origanum vulgare* (6), *Potentilla neumanniana* (6), *Ranunculus bulbosus* (6), *Thymus pulegioides* (6), *Cirsium arvense* (5), *Eupatorium cannabinum* (5), *Knautia arvensis* (5) and *Picris hieracioides* (5) |

**Considered as a module hub between 1990 and 2009 if we consider the 95% quantiles of *cz*-coefficients of null models.
